# High prevalence and clinical impact of microbial co-detection in hospitalized children with human parainfluenza virus type 1

**DOI:** 10.1128/spectrum.00275-26

**Published:** 2026-06-16

**Authors:** Chunyun Fu, Xiaoying Chen, Lishai Mo, Junming Lu, Ya Huang, Jiangyang Zhao, Yuanyuan He, Juan Lin, Yanqing Tang, Shang Yi, Hao Wei, Xuehua Hu, Jie Tan

**Affiliations:** 1 Medical Science Laboratory, Children’s Hospital, Maternal and Child Health Hospital of Guangxi Zhuang Autonomous Region, Nanning, People’s Republic of China; 2 Department of Pediatric Respiratory Medicine, Children’s Hospital, Maternal and Child Health Hospital of Guangxi Zhuang Autonomous Region, Nanning, People’s Republic of China; 3 Department of Genetic Metabolism, Children’s Hospital, Maternal and Child Health Hospital of Guangxi Zhuang Autonomous Region, Nanning, People’s Republic of China; Tainan Hospital Ministry of Health and Welfare, Tainan, Taiwan; Wasit University, Kut, Wasit, Iraq

**Keywords:** human parainfluenza virus type 1, acute respiratory infection, tNGS, co-detection, children

## Abstract

Human parainfluenza virus type 1 (HPIV-1) is an important but underrecognized pathogen associated with acute respiratory infections (ARI) in children, although its clinical impact remains poorly defined, particularly with microbial co-detection. This study enrolled hospitalized children with ARI who underwent targeted next-generation sequencing (tNGS). Clinical features were compared between HPIV-1 mono-detection and co-detection groups, as well as between patients with severe and non-severe pneumonia. Among 9,394 enrolled children, HPIV-1 was detected in 1.7%, predominantly in those aged 6 months to 2 years. Co-detection with other respiratory pathogens was observed in 95.7% of HPIV-1-positive cases, with viral-bacterial co-infection being the most common pattern. *Haemophilus influenzae*, *Streptococcus pneumoniae*, rhinovirus, and *Mycoplasma pneumoniae* were the most frequently co-detected agents. Compared to the mono-detection group (mostly infants ≤12 months), children with co-detection were older, exhibited higher total immunoglobulin levels, experienced longer hospitalizations, and incurred greater treatment costs (all *P* < 0.05). Among HPIV-1-positive children, respiratory and non-respiratory complications occurred in approximately one-third and two-fifths of cases, respectively, with over 95% achieving clinical improvement or discharge. Patients with severe pneumonia, compared to non-severe cases, demonstrated significantly elevated inflammatory markers, prolonged fever, higher rates of complications and ICU admission, increased respiratory support requirements, longer hospital stays, and higher costs (all *P* < 0.05). HPIV-1-positive pediatric ARI patients have a high co-detection rate, with HPIV-1-virus-bacteria co-detection being the predominant pattern. Co-detection is linked to greater disease severity compared to mono-detection. Key indicators for severe pneumonia include elevated inflammatory markers, prolonged fever, complications, and respiratory support needs.

**IMPORTANCE:**

Human parainfluenza virus type 1 (HPIV-1) is an underrecognized contributor to pediatric acute respiratory infections (ARI), yet its clinical impact remains unclear, particularly regarding co-detection with other microbes. Based on targeted next-generation sequencing (tNGS), this study demonstrates that HPIV-1 detection in hospitalized children with ARI is characterized by a high rate of microbial co-detection, which is associated with greater disease severity. Elevated inflammatory markers (procalcitonin [PCT] and erythrocyte sedimentation rate [ESR]), prolonged fever, complication occurrence, and need for respiratory support serve as key predictors of progression to severe pneumonia in these children. These findings offer important clinical evidence to support the development of a predictive model for severe pneumonia in HPIV-1-positive children and provide valuable guidance for optimizing diagnostic and therapeutic strategies for HPIV-1-associated respiratory infections.

## INTRODUCTION

Acute respiratory infections (ARI) are one of the leading causes of hospitalization and mortality in children under 5 years of age worldwide (1). Viruses are the most common cause of pediatric respiratory infections (2). According to the first global burden estimates for acute lower respiratory infections in children, human parainfluenza virus (HPIV)—an enveloped, negative-sense single-stranded RNA virus belonging to the family Paramyxoviridae—is responsible for approximately 13% of all pediatric acute lower respiratory infection cases and is associated with 4% to 14% of related hospitalizations (1), thereby contributing substantially to the healthcare burden. HPIV comprises four serotypes (HPIV-1 to HPIV-4), with HPIV-1 being associated with the largest and most well-documented outbreaks (3, 4). In young children, HPIV-1 is particularly known to cause severe clinical manifestations, such as laryngotracheobronchitis, bronchiolitis, and pneumonia, and may also compromise immune function (3, 5–7), thereby posing significant challenges in clinical management. As a result, HPIV-1 has been recognized as an important pathogen in children hospitalized with ARI.

Despite the established role of HPIV-1 as a significant pediatric pathogen, critical gaps remain in two key areas. First, technical limitations persist. Most studies have relied on quantitative real-time PCR (8, 9). Though specific, this method may fail to detect novel strains or cases with low viral loads due to its targeted nature, potentially leading to an underestimation of the detection rate. Second, the prevalence, patterns, and clinical impact of HPIV-1 co-detection with other agents remain poorly characterized (8, 9).

This study utilized targeted next-generation sequencing (tNGS)—a method chosen for its sensitivity and broad pathogen coverage (10, 11)—to screen over 10,000 pediatric ARI inpatients. It aimed to investigate the epidemiology, co-detection patterns, and clinical features of HPIV-1-positive children. Comparisons were made between the mono-detection and microbial co-detection groups, and between severe and non-severe pneumonia cases. The results are expected to inform the clinical management of HPIV-1-associated disease in children.

## MATERIALS AND METHODS

### Study population

This retrospective study analyzed 10,153 pediatric patients hospitalized with ARI at the Maternal and Child Health Hospital of Guangxi Zhuang Autonomous Region between April 2021 and December 2024. Clinical information and laboratory data were collected for all included cases. ARI is defined as an acute clinical condition resulting from various pathogens, characterized by the sudden onset of respiratory symptoms and typically lasting no more than 21 days. Common clinical manifestations include cough, sputum production, shortness of breath, sore throat, and rhinorrhea. The spectrum of ARI encompasses acute upper respiratory tract infections, acute bronchitis, and community-acquired pneumonia.

### Definition of severe and non-severe pneumonia

Severe pneumonia was defined as radiologically confirmed pneumonia meeting at least one of the following major criteria: (i) general danger signs (e.g., impaired consciousness, convulsions, inability to feed, dehydration, or severe vomiting); (ii) respiratory distress or hypoxemia (e.g., central cyanosis, SpO₂ ≤ 92% on room air, grunting, severe tachypnea, or chest wall indrawing); (iii) respiratory failure necessitating non-invasive or invasive mechanical ventilation; (iv) radiological evidence of complications (e.g., multi-lobar involvement, pleural effusion, or necrotizing pneumonia); (v) systemic complications (e.g., hemodynamic instability, shock, heart failure, acute kidney injury, or other organ dysfunction).

For this study, organ dysfunction was defined as meeting one or more of the following criteria: (i) cardiovascular dysfunction: requiring vasoactive agents or presenting with septic shock; (ii) neurological dysfunction: Glasgow Coma Score ≤11 or a rapid decline of ≥3 points; (iii) renal dysfunction: serum creatinine ≥2 times the age-specific upper limit of normal or requiring renal replacement therapy; (iv) hepatic dysfunction: hyperbilirubinemia, coagulopathy, or transaminase elevation >5 times the upper limit of normal; (v) hematological dysfunction: disseminated intravascular coagulation or severe thrombocytopenia. Notably, isolated mild transaminase elevations (≤5 times the upper limit of normal) without other hepatic impairment were excluded from this classification.

Non-severe pneumonia was diagnosed in children with compatible clinical symptoms and radiological evidence of pneumonia, provided that none of the severe pneumonia criteria were met. These patients typically presented with fever, cough, and tachypnea, but without signs of severe respiratory distress, hypoxemia, or major systemic complications.

### Inclusion and exclusion criteria

Included cases had to meet all the following criteria: aged ≤14 years, hospitalized due to an acute respiratory tract infection (12–15), and having undergone tNGS testing with available results. Cases were excluded for any of the following reasons: incomplete essential clinical or laboratory data (e.g., missing demographics, tNGS reports, or clinical diagnosis records); a confirmed non-infectious etiology for respiratory symptoms (e.g., allergies, asthma, or environmental irritants); or a diagnosis of hospital-acquired infection.

### Targeted next-generation sequencing

A total of 10,153 clinical specimens—including 4,578 bronchoalveolar lavage fluid samples, 4,648 throat swabs, and 927 sputum samples—were collected under standardized clinical protocols (16). Total nucleic acid extraction was performed using the MagPure Pathogen DNA/RNA Kit (R6672-01B, Magen, Guangzhou, China). Subsequent library preparation for tNGS was performed with the Respiration100 multiplex PCR system (KingCreate, Guangzhou, China) to detect common respiratory pathogens, including HPIV-1. It should be noted that the manufacturer’s panel was updated during the study period, leading to a significant expansion of the detection spectrum from an initial 65 to a final 198 targeted agents (Tables S1 and S2). During the COVID-19 pandemic phase, all children hospitalized with ARI were required to undergo routine SARS-CoV-2 RT-PCR testing upon admission. Only those with negative RT-PCR results who also met clinical indications for tNGS were enrolled for tNGS testing. The major analytical platforms utilized for tNGS data processing and analysis were the DRAGEN Bio-IT Platform, Galaxy, and CLC Genomics Workbench. A comprehensive description of the tNGS methodology and workflow has been published previously (17). The interpretation of targeted sequencing results relied on two key metrics: amplicon coverage and normalized read count. Detection of microbes was defined according to the following criteria: (i) for bacteria (excluding the *Mycobacterium tuberculosis* complex), fungi, and atypical pathogens: amplicon coverage ≥50% and normalized read count ≥10; (ii) for viruses: amplicon coverage ≥50% and normalized read count ≥3, or a normalized read count ≥10; (iii) for the *Mycobacterium tuberculosis* complex: a normalized read count ≥1 was considered indicative of detection.

### Definition of infection versus colonization

Subsequently, two experienced clinicians independently evaluated the clinical data to determine the relevance of potential pathogens to the ARI and overall clinical presentation. Their comprehensive assessment incorporated the patient’s medical history, clinical symptoms, results of routine microbiological tests, inflammatory markers, epidemiological features, imaging findings, tNGS results, and other laboratory data. In cases of disagreement, a senior consultant was consulted to reach a consensus. To ensure diagnostic accuracy and systematically differentiate true pathogenic infection from colonization, this evaluation was guided by a multidimensional framework that assessed four clinical domains: (i) host factors (e.g., immunosuppression due to neutropenia, HIV, transplantation, or prematurity); (ii) microbiological evidence (e.g., elevated pathogen burden via tNGS, low viral PCR Ct values, invasive forms on microscopy, or positive cultures from sterile sites); (iii) clinical and radiographic features (e.g., new or progressive infiltrates, ground-glass opacities, viral patterns on imaging, or elevated markers, such as procalcitonin, C-reactive protein, galactomannan, or virus-specific antibodies); and (iv) therapeutic response (e.g., clinical improvement within 72 hours of targeted therapy or confirmed virological clearance).

### Statistical analysis

Statistical analyses were performed using IBM SPSS Statistics version 26.0. Measurement data conforming to a normal distribution with homogeneity of variance are presented as the mean ± standard deviation ($\bar{x}$ ± s), and comparisons between two groups were conducted using the independent samples *t*-test. Data not following a normal distribution are expressed as the median (interquartile range), and the Mann-Whitney *U* test was used for comparisons between two groups of non-parametric data. Categorical data are presented as frequency (percentage, %), and intergroup comparisons were made using the chi-square (χ²) test. The goodness-of-fit chi-square test analyzed distribution differences. *P* < 0.05 was considered statistically significant.

## RESULTS

### Detection rate of HPIV-1

**Fig 1:**
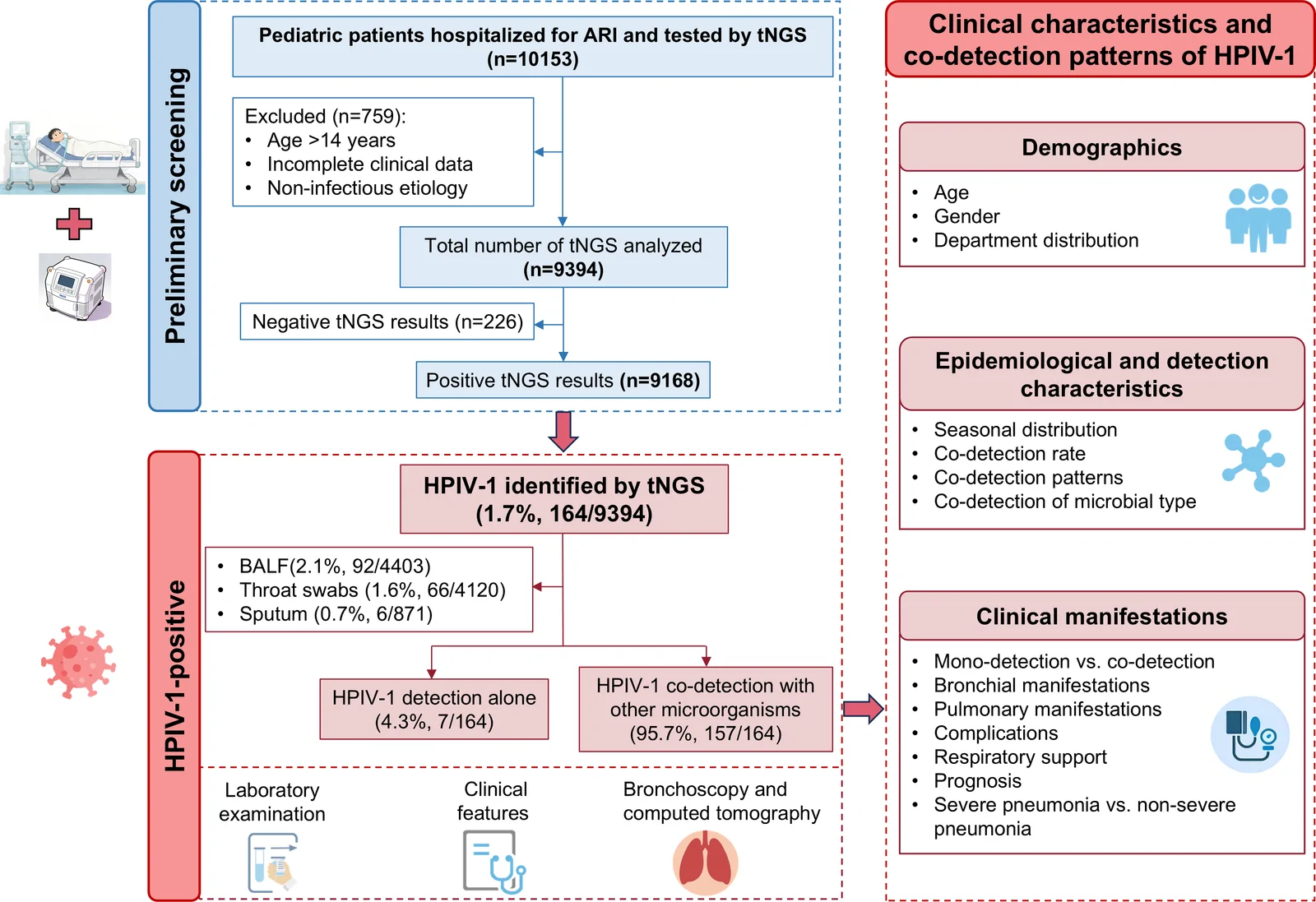
Study flow diagram: cohort selection, tNGS testing, and HPIV-1 analysis in children with acute respiratory infections. ARI, acute respiratory infection; tNGS, targeted next-generation sequencing; HPIV-1, human parainfluenza virus 1; BALF, bronchoalveolar lavage fluid.

Among the 10,153 hospitalized children with ARI and tested tNGS, a total of 9,394 cases met the inclusion criteria for the study (Fig. 1). Among them, 226 cases had negative tNGS results, and 9,168 cases had positive tNGS results, with a positive rate of 97.6% (9,168/9,394). A total of 164 cases were detected with HPIV-1, with a detection rate of 1.7% (164/9,394). In terms of specimen types, the detection rate of HPIV-1 was the highest in bronchoalveolar lavage fluid (2.1%, 92/4,403), followed by throat swabs (1.6%, 66/4,120) and sputum (0.7%, 6/871).

### Demographics of HPIV-1-positive children with ARI

The 164 HPIV-1-positive children showed an uneven age distribution (*P* < 0.001), with cases concentrated in the 0.5–1 year (20.7%) and 1–2 years (22.6%) age groups; the median age was 20.0 months (IQR: 10.0–45.8 months). The gender distribution among these children (61.0% male, 39.0% female) was consistent with that of the overall study population (60.1% male, 39.9% female). The distribution across hospital admission departments was also significantly different (*P* < 0.001). The majority were admitted to the Pediatric Respiratory Department (58.5%), followed by the Pediatric Cardiology Department (15.2%); the remaining cases were distributed among other departments. Detailed demographic information is presented in Table 1.

**TABLE 1:** Demographic characteristics of children with HPIV-1-associated acute respiratory infection^*a*^

Characteristics	Patients infected with HPIV-1 (*n* = 164)	*P* value^*b*^
Age		
Median age (months), (IQR)	20.0 (10.0, 45.8)	
≤3 months, *n* (%)	12 (7.3%)	**<0.001**
3–6 months, *n* (%)	13 (7.4%)	
6–12 months, *n* (%)	34 (20.7%)	
1–2 years, *n* (%)	37 (22.6%)	
2–3 years, *n* (%)	15 (9.2%)	
3–5 years, *n* (%)	32 (19.5%)	
5–12 years, *n* (%)	21 (12.8%)	
Gender		
Male, *n* (%)	100 (61.0%)	**<0.01**
Female, *n* (%)	64 (39.0%)	
Department		
Pediatric Respiratory Medicine	96 (58.5%)	**<0.001**
Pediatric Cardiology	25 (15.2%)	
Pediatric Intensive Care Unit	11 (6.7%)	
Pediatric Infectious Diseases	8 (4.9%)	
Pediatric Nephrology	7 (4.3%)	
Pediatric Gastroenterology	7 (4.3%)	
Pediatric Neurology	6 (3.7%)	
Pediatric General Surgery	2 (1.2%)	
Neonatology	1 (0.6%)	
Pediatric Otolaryngology	1 (0.6%)	

^*a*^ HPIV-1, human parainfluenza virus type 1; IQR, interquartile range.

^*b*^ Values in bold indicate statistical significance (*P *< 0.05).

### Epidemiological and detection characteristics of HPIV-1 in hospitalized children with ARI

**Fig 2:**
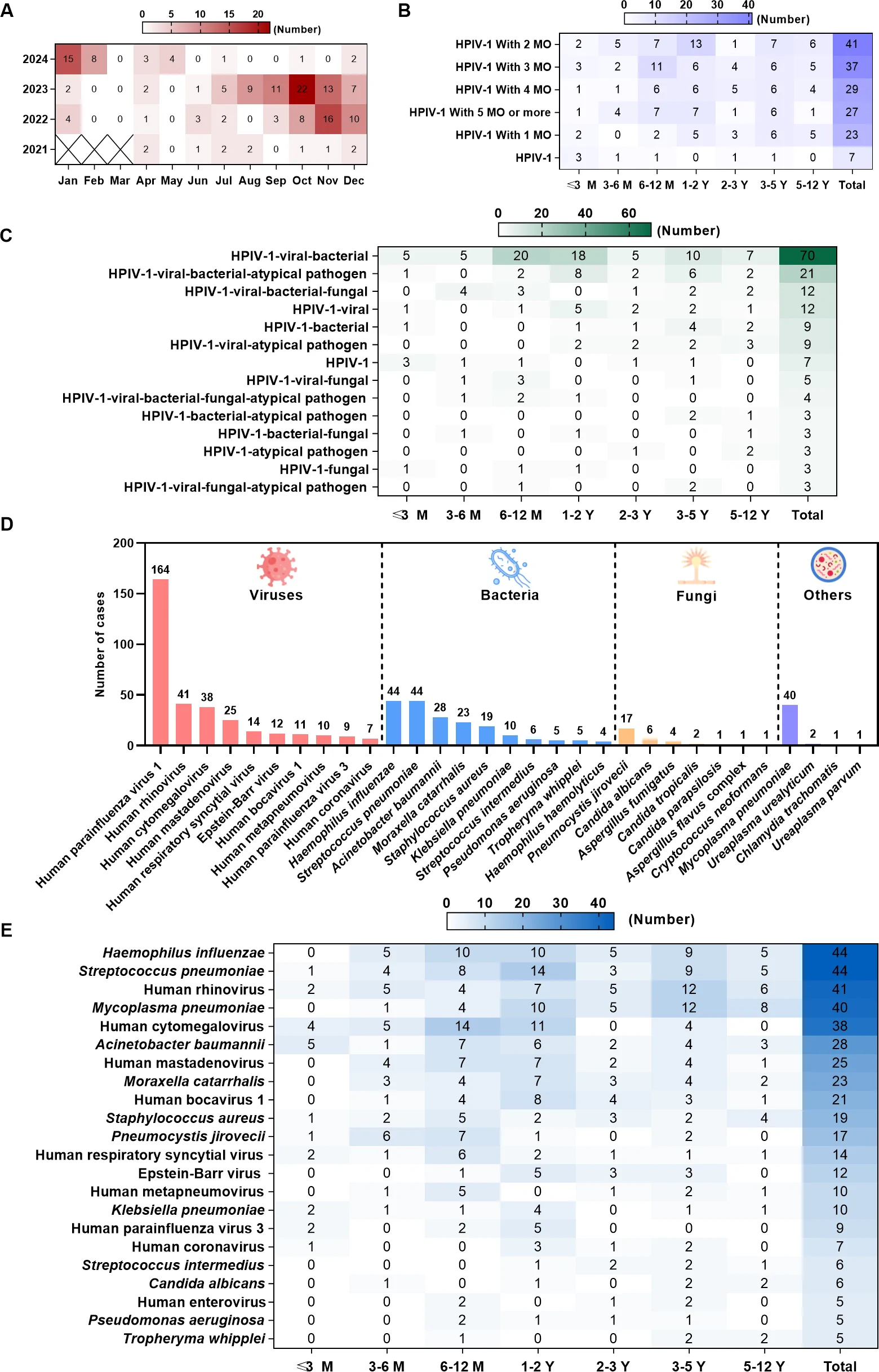
Epidemiological and co-detection profiles of HPIV-1 in pediatric acute respiratory infection. (**A**) Monthly HPIV-1 detections by tNGS (April 2021 to December 2024). (**B**) Distribution of the number of co-detected targeted respiratory agents by age group. (**C**) Distribution of co-detection patterns by age group. (**D**) The main microbes were detected in 164 HPIV-1-positive children. (**E**) Top 22 co-detected targeted respiratory agents by age group. HPIV-1, human parainfluenza virus type 1; MO, microbes; M, months; Y, years.

From April 2021 to December 2024, 164 children hospitalized with ARI were positive for HPIV-1. Cases exhibited a distinct seasonal distribution, predominantly in autumn and winter, with peaks in November 2022, October 2023, and January 2024 (Fig. 2A). Mono-detection was uncommon (*n* = 7, 4.3%). Most cases (*n* = 157, 95.7%) involved co-detection (Fig. 1 and 2B), most frequently with two (*n* = 41, 25.0%) or three (*n* = 37, 22.6%) other targeted respiratory agents (Fig. 2B). The predominant compositional pattern was HPIV-1-virus-bacteria co-detection (*n* = 70, 42.7%), followed by HPIV-1-virus-bacteria-atypical agent co-detection (*n* = 21, 12.8%) (Fig. 2C). tNGS identified 53 distinct targeted respiratory agents co-detected with HPIV-1, comprising 18 viruses, 24 bacteria, 7 fungi, and 4 atypical agents (Fig. 2D). The primary age distribution of the top five co-detected agents was as follows: *Haemophilus influenzae* and *Streptococcus pneumoniae* (both most common in children aged 6 months to 2 years, and 3–5 years), human rhinovirus (3–5 years), *Mycoplasma pneumoniae* (1–2 years and 3–5 years), and human cytomegalovirus (6 months to 2 years) (Fig. 2E). In contrast, children with HPIV-1 mono-detection were predominantly aged ≤12 months (5 out of 7, 71.4%) (Fig. 2B and C). To assess potential bias arising from variable panel size over time, we performed a sensitivity analysis restricted to a core panel of 64 uniformly screened targets. This analysis yielded similar overall co-detection rates compared to the original full-panel analysis: 94.5% versus 95.7%, with an absolute difference of only 1.2%. Since HPIV-1 was present in all panel versions, its detection rate remained unchanged in this sensitivity analysis.

### Features of HPIV-1 mono-detection and co-detection

Clinical and laboratory features of the HPIV-1 mono-detection (*n* = 7) and co-detection (*n* = 157) groups are presented in Table 2. Children with co-detection were significantly older (median 21.0 vs 6.0 months, *P* = 0.019). They also exhibited significantly lower cystatin C (0.9 vs 1.1 mg/L, *P* = 0.015) and higher levels of total IgG (8.0 vs 6.4 g/L), total IgA (0.6 vs 0.2 g/L), and total IgM (1.2 vs 0.8 g/L) (all *P* < 0.05). Clinically, the co-detection group had longer hospitalizations (7.0 vs 5.0 days, *P* = 0.033) and higher treatment costs (8,912.4 vs 4,142.3 CNY, *P* = 0.030). A stratified analysis based on the top eight co-detected microbes yielded results largely consistent with the overall co-detection group (Table S3).

**TABLE 2:** Comparison of laboratory parameters and clinical features between children with HPIV-1 mono-detection and those with microbial co-detection^*a*^

Characteristics	HPIV-1 mono-detection (*n* = 7)	HPIV-1 co-detection (*n* = 157)	*P* value^*b*^
Quantitative data (median [IQR]) or (mean ± SD)			
Age (months)	6.0 (1.1, 25.0)	21.0 (10.9, 46.5)	**0.019**
RBC (×10^12^/L)	4.3 (3.8, 4.7)	4.6 (4.3, 5.0)	0.180
WBC (×10^9^/L)	7.8 (6.3, 9.8)	9.1 (6.7, 11.6)	0.566
NEU (%)	36.7 ± 21.8	47.0 ± 18.9	0.162
LYM (%)	51.0 ± 19.7	42.7 ± 17.5	0.222
Mon (%)	9.0 (8.5, 13.3)	8.0 (5.6, 10.8)	0.219
Eos (%)	1.5 (0.1, 3.5)	0.7 (0.1, 1.6)	0.509
Baso (%)	0.2 (0.0, 0.2)	0.2 (0.1, 0.4)	0.106
PLT (×10^9^/L)	397.0 (307.0, 498.0)	338.0 (265.5, 448.0)	0.251
CRP (mg/L)	1.6 (0.5, 22.6)	2.8 (0.6, 7.8)	0.667
PCT (ng/mL)	0.1 (0.1, 0.3)	0.1 (0.1, 0.2)	0.471
AST (U/L)	35.0 (31.0, 105.0)	37.0 (31.0, 47.5)	0.684
ALT (U/L)	22.0 (14.0, 59.0)	16.0 (12.0, 26.0)	0.160
ESR (mm/h)	17.0 (15.0, 26.0)	20.0 (13.0, 33.5)	0.720
D-dimer (ng/mL FEU)	449.0 (360.0, 1,270.0)	430.0 (292.0, 750.0)	0.489
LDH (U/L)	323.0 (293.0, 424.0)	313.0 (276.0, 351.5)	0.251
URE (mmol/L)	20.0 (15.0, 30.0)	26.0 (20.0, 32.0)	0.153
CYs-C (mg/L)	1.1 (1.0, 1.6)	0.9 (0.8, 1.1)	**0.015**
CK (U/L)	66.0 (53.0, 86.0)	88.0 (60.5, 125.0)	0.078
CK-MB (U/L)	27.0 (23.0, 36.0)	26.0 (20.0, 31.5)	0.336
IgG (g/L)	6.4 (4.1, 6.7)	8.0 (6.4, 9.6)	**0.014**
IgA (g/L)	0.2 (0.2, 0.6)	0.6 (0.3, 1.0)	**0.047**
IgM (g/L)	0.8 (0.4, 1.1)	1.2 (0.8, 1.7)	**0.014**
Duration of fever (days)	2.0 (0.0, 2.0)	2.0 (0.0, 3.0)	0.334
Length of hospitalization (days)	5.0 (4.0, 6.0)	7.0 (5.0, 10.0)	**0.033**
Treatment expense (CNY)	4,142.3 (2,773.6, 8,408.5)	8,912.4 (5,754.7, 12,277.3)	**0.030**
Categorical data (n [%])			
Gender (male)	3 (42.9%)	97 (61.8%)	0.543
Fever	4 (57.1%)	113 (72.0%)	0.673
Intensive care unit (ICU) admission	0 (0.0%)	11 (7.0%)	1.000
Bronchitis	3 (42.7%)	69 (44.0%)	1.000
Bilateral pneumonia	2 (28.6%)	53 (33.8%)	1.000
Unilateral pneumonia	1 (14.3%)	12 (7.6%)	1.000
Bronchopneumonia	0 (0.0%)	5 (3.2%)	1.000
Severe pneumonia	0 (0.0%)	16 (10.2%)	1.000
Respiratory complications	1 (14.3%)	58 (36.9%)	0.412
Non-respiratory complications	2 (28.6%)	64 (40.8%)	0.803
Respiratory support	0 (0.0%)	28 (17.8%)	0.604
Invasive respiratory support	0 (0.0%)	13 (8.3%)	1.000
Discharged with improvement	7 (100.0%)	149 (94.9%)	1.000

^*a*^ HPIV-1, human parainfluenza virus type 1; IQR, interquartile range; SD, standard deviation; RBC, red blood cell; WBC, white blood cell; NEU, neutrophil; LYM, lymphocyte; Mon, monocyte; Eos, eosinophil; Baso, basophil; PLT, platelet; CRP, C-reactive protein; PCT, procalcitonin; AST, aspartate aminotransferase; ALT, alanine aminotransferase; ESR, erythrocyte sedimentation rate; LDH, lactate dehydrogenase; URE, urea; CYs-C, cystatin C; CK, creatine kinase; CK-MB, creatine kinase-MB.

^*b*^ Values in bold indicate statistical significance (*P *< 0.05).

### Age-stratified and specimen type analyses

To address potential age-related bias in specimen collection and co-detection patterns, we performed stratified analyses by age group and specimen type (Tables S4 and S5). As shown in Table S4, BALF was more frequently collected in children aged >12 months than in those ≤12 months (62.9% vs 44.1%, *P* = 0.020). However, the detection rates of the three most common co-detected pathogens—*H*. *influenzae*, *S. pneumoniae*, and human rhinovirus—were comparable across BALF, throat swab, and sputum specimens (all *P* > 0.05), indicating minimal specimen type bias in co-detection analysis.

In the age-stratified subgroup analysis (Table S5), among children ≤12 months (mono-detection: *n* = 5; co-detection: *n* = 54), co-detection was associated with significantly longer hospital stays (7.0 vs 5.0 days, *P* = 0.006) and higher treatment expenses (9,194.1 vs 3,358.0 CNY, *P* = 0.009). In contrast, among children >12 months (mono-detection: *n* = 2; co-detection: *n* = 98), no significant differences were observed in these outcomes (*P* > 0.05), likely due to the extremely small mono-detection sample size in this subgroup. These findings suggest that the association between HPIV-1 co-detection and increased disease severity is independent of age, particularly in infants and young children.

### Bronchoscopy and computed tomography findings

**Fig 3:**
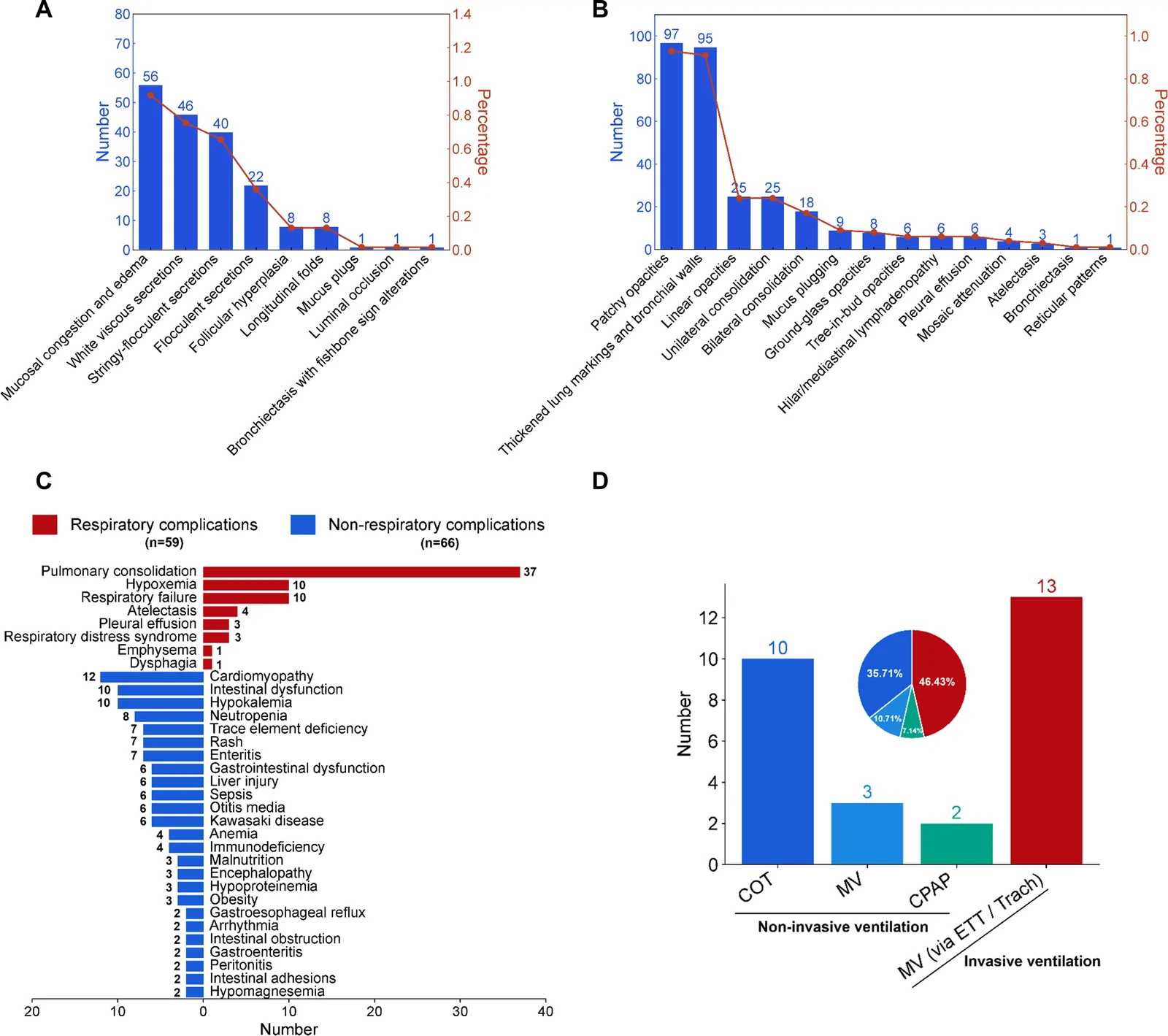
Clinical examinations, complications, and respiratory support in children with HPIV-1 acute respiratory infection. (**A**) Bronchoscopic findings (*n* = 61). (**B**) Chest computed tomography findings (*n* = 104). (**C**) Spectrum of respiratory (*n* = 59) and non-respiratory (*n* = 66) complications. (**D**) Utilization of non-invasive and invasive respiratory support (*n* = 28). HPIV-1, human parainfluenza virus type 1; COT, central oxygen therapy; MV, mechanical ventilation; CPAP, continuous positive airway pressure; MV (via ETT/Trach), mechanical ventilation via endotracheal tube/tracheostomy.

Of the 164 HPIV-1-positive children, 61 (37.2%) underwent bronchoscopy (Fig. 3A). The main abnormalities observed were mucosal congestion and edema (*n* = 56, 91.8%), white viscous mucosal secretions (*n* = 46, 75.4%), strip- or floc-like secretions in lavage fluid (*n* = 40, 65.6%), and wool-like secretions (*n* = 22, 36.1%).

Chest CT was performed in 104 children (63.4%) (Fig. 3B). The predominant abnormal findings included patchy opacities (*n* = 97, 93.3%), thickening of lung markings and bronchial walls (*n* = 95, 91.3%), linear opacities (*n* = 25, 24.0%), unilateral consolidation (*n* = 25, 24.0%), and bilateral consolidation (*n* = 18, 17.3%).

### Complications and respiratory support

Of the 164 children with HPIV-1-associated ARI, 59 (36.0%) developed respiratory complications, primarily pulmonary consolidation (*n* = 37, 62.7%), hypoxia (*n* = 10, 17.0%), and respiratory failure (*n* = 10, 17.0%) (Fig. 3C). Non-respiratory complications occurred in 66 children (40.24%), mainly including cardiomyopathy (*n* = 12, 18.2%), intestinal dysfunction (*n* = 10, 15.2%), hypokalemia (*n* = 10, 15.2%), and neutropenia (*n* = 8, 12.1%) (Fig. 3C).

Respiratory support was required in 28 cases (17.1%). Non-invasive respiratory support was provided to 15 children (53.6% of those requiring support), comprising supplemental oxygen therapy (*n* = 10, 66.8%), non-invasive ventilation (*n* = 3, 20.0%), and continuous positive airway pressure (CPAP, *n* = 2, 13.3%). Invasive respiratory support was administered to 13 children (46.4%), all via endotracheal intubation or tracheostomy coupled with invasive mechanical ventilation (Fig. 3D).

### Prognosis

Of the 164 children with HPIV-1-associated ARI, the primary clinical diagnosis was bronchitis (43.9%, 72/164), followed by bilateral pneumonia (33.5%, 55/164) and unilateral pneumonia (7.9%, 13/164). Severe pneumonia was diagnosed in 16 children (9.8%), 11 of whom required ICU admission. The median hospital stay was 7.0 days (IQR: 5.0–10.0 days), with a median treatment cost of 8,761.7 CNY (IQR: 5,403.5–12,094.5 CNY). Following standard symptomatic treatment, 156 children (95.1%) improved and were discharged. The remaining eight cases comprised two discharges against medical advice (due to family refusal of treatment) and six voluntary discharges (attributed to perceived insufficient efficacy or financial constraints).

### Comparison of clinical features between severe and non-severe pneumonia in children with HPIV-1

Clinical features differed significantly between children with severe and non-severe HPIV-1-associated pneumonia (Table 3). Compared with the non-severe group, the severe group had higher levels of procalcitonin (PCT) (0.5 vs 0.1 ng/mL, *P* < 0.001) and erythrocyte sedimentation rate (ESR) (38.00 vs 20.00 mm/h, *P* = 0.007), longer duration of fever (7.0 vs 2.0 days, *P* < 0.001) and hospital stay (17.5 vs 6.0 days, *P* < 0.001), and higher treatment costs (57,725.5 vs 8,357.1 CNY, *P* < 0.001). The severe group had significantly higher rates of bilateral pneumonia (68.8% vs 29.7%, *P* = 0.002), ICU admission (68.8% vs 0.0%, *P* < 0.001), respiratory complications (93.8% vs 29.7%, *P* < 0.001), and non-respiratory complications (81.3% vs 35.8%, *P* = 0.001). Additionally, the severe group more frequently required respiratory support (87.5% vs 9.5%, *P* < 0.001) and invasive respiratory support (68.8% vs 1.4%, *P* < 0.001), and had a lower rate of favorable discharge (81.3% vs 96.6%, *P* < 0.001). Although statistically significant differences were noted in measures, such as white blood cell count (WBC) and neutrophil percentage (NEU%), the median/mean values for all these parameters fell within normal reference ranges.

**TABLE 3:** Clinical characteristics of children with HPIV-1-associated pneumonia: severe versus non-severe cases^*a*^

Characteristics	Severe pneumonia (*n* = 16)	Non-severe pneumonia (*n* = 148)	*P* value^*b*^
Quantitative data (median [IQR]) or (mean ± SD)			
Age (months)	20.5 (8.3, 75.0)	20.0 (10.0, 45.0)	0.874
RBC (×10^12^/L)	4.2 (3.5, 5.3)	4.6 (4.3, 4.9)	0.204
WBC (×10^9^/L)	9.7 (8.4 14.6)	8.9 (6.4, 11.3)	**0.048**
NEU (%)	62.8 ± 18.8	44.8 ± 18.4	**<0.001**
LYM (%)	29.8 ± 17.5	44.5 ± 17.1	**0.001**
Mon (%)	5.6 (4.6, 9.1)	8.4 (6.4, 11.0)	**0.024**
Eos (%)	0.1 (0.0, 0.9)	0.7 (0.2, 1.8)	**0.002**
Baso (%)	0.2 (0.1, 0.3)	0.2 (0.1, 0.4)	0.384
PLT (×10^9^/L)	360.5 (267.8, 431.5)	341.0 (267.5, 450.5)	0.794
CRP (mg/L)	4.3 (1.2, 28.2)	2.7 (0.5, 7.2)	0.151
PCT (ng/mL)	0.5 (0.1, 3.5)	0.1 (0.1, 0.2)	**<0.001**
AST (U/L)	39.5 (28.3, 53.0)	37.0 (31.0, 47.8)	0.784
ALT (U/L)	19.0 (13.3, 47.8)	16.5 (12.0, 26.0)	0.284
ESR (mm/h)	38.0 (15.5, 55.0)	20.0 (13.0, 29.0)	**0.007**
D-dimer (ng/mL FEU)	785.0 (270.0, 11,307.5)	430.0 (302.5, 672.5)	0.172
LDH (U/L)	344.0 (268.5, 381.0)	308.5 (277.3, 348.0)	0.186
URE (mmol/L)	27.0 (22.0, 41.0)	25.0 (19.2, 32.0)	0.286
CYs-C (mg/L)	1.0 (0.8, 1.5)	0.9 (0.8, 1.1)	0.168
CK (U/L)	60.0 (45.0, 97.5)	89.0 (64.0, 125.0)	**0.031**
CK-MB (U/L)	25.0 (17.5, 30.0)	26.0 (21.0, 32.0)	0.293
IgG (g/L)	7.1 (4.9, 10.3)	8.0 (6.5, 9.4)	0.490
IgA (g/L)	0.8 (0.2, 1.8)	0.6 (0.3, 1.0)	0.610
IgM (g/L)	1.2 (0.9, 2.0)	1.2 (0.8, 1.6)	0.624
Duration of fever (days)	7.0 (1.5, 18.0)	2.0 (0.0, 3.0)	**<0.001**
Length of hospitalization (days)	17.5 (9.5, 24.5)	6.0 (5.0, 9.0)	**<0.001**
Treatment expense (CNY)	57,725.5 (18,936.8, 98,507.9)	8,357.1 (5,117.9, 10,688.9)	**<0.001**
Categorical data (*n,* %)			
Gender (male)	11 (68.8%)	89 (60.1%)	0.502
Fever	14 (87.5%)	103 (69.6%)	0.225
Patients with HPIV-1 co-infection	0 (0.0%)	7 (4.7%)	1.000
Bronchitis	5 (31.3%)	67 (45.3%)	0.283
Bilateral pneumonia	11 (68.8%)	44 (29.7%)	**0.002**
Unilateral pneumonia	0 (0.0%)	13 (8.8%)	0.368
Bronchopneumonia	0 (0.0%)	5 (3.4%)	1.000
Intensive care unit (ICU) admission	11 (68.8%)	0 (0.0%)	**<0.001**
Respiratory complications	15 (93.8%)	44 (29.7%)	**<0.001**
Non-respiratory complications	13 (81.3%)	53 (35.8%)	**0.001**
Respiratory support	14 (87.5%)	14 (9.5%)	**<0.001**
Invasive respiratory support	11 (68.8%)	2 (1.4%)	**<0.001**
Discharged with improvement	13 (81.3%)	143 (96.6%)	**0.036**

^*a*^ HPIV-1, human parainfluenza virus type 1; IQR, interquartile range; SD, standard deviation; RBC, red blood cell; WBC, white blood cell; NEU, neutrophil; LYM, lymphocyte; Mon, monocyte; Eos, eosinophil; Baso, basophil; PLT, platelet; CRP, C-reactive protein; PCT, procalcitonin; AST, aspartate aminotransferase; ALT, alanine aminotransferase; ESR, erythrocyte sedimentation rate; LDH, lactate dehydrogenase; URE, urea; CYs-C, Cystatin C; CK, creatine kinase; CK-MB, creatine kinase-MB.

^*b*^ Values in bold indicate statistical significance (*P *< 0.05).

## DISCUSSION

HPIV-1 is an important etiological agent of ARI in children. While previous studies have described the epidemiology and genetic features of HPIV-1 in pediatric populations (6, 18), none have systematically analyzed its epidemiology, microbial co-detection profiles, and associated clinical characteristics using tNGS in a large cohort of hospitalized children with ARI. To address this gap, our study enrolled 10,153 hospitalized ARI children tested by tNGS (9,394 meeting inclusion criteria) and demonstrated a high rate of microbial co-detection with HPIV-1. Furthermore, we identified early-warning indicators for severe pneumonia in children with HPIV-1-associated illness, offering clinically useful guidance.

Compared with conventional methods, tNGS enables more comprehensive microbial profiling by specifically enriching target nucleic acids through techniques such as primer amplification or hybridization capture (11). In this study, tNGS detection revealed an overall HPIV-1 positivity rate of 1.7% among 9,394 hospitalized children with ARI, with the highest rate (2.1%) observed in bronchoalveolar lavage fluid. Affected children were predominantly male (61.0%) and aged 6 months to 2 years, with a higher frequency of detection in autumn and winter—findings that align with previously reported epidemiological trends for HPIV-1 (3, 19). However, it should be noted that the typical biennial odd-year peak pattern of HPIV-1 was not observed in this study, which may be related to the impact of the COVID-19 pandemic on respiratory virus circulation during the study period (2021–2024) (20, 21). A key finding was the high rate of microbial co-detection with HPIV-1: up to 95.7% of HPIV-1-positive children had other microbes detected concurrently, most commonly in an HPIV-1-virus-bacteria pattern. This rate substantially exceeds those reported in studies using traditional detection methods (22, 23); for example, one multiplex PCR-based study reported a co-detection rate of 24.8% for multiple respiratory pathogens (24), underscoring the capacity of tNGS to better reveal the complex microbial landscape in respiratory illnesses. Among co-detected microbes, *Haemophilus influenzae*, *Streptococcus pneumoniae*, Rhinovirus, and *Mycoplasma pneumoniae* were the most frequent (each >25.0%). It is important to note that not all co-detected microbes are necessarily causative pathogens; some may represent colonization, residual nucleic acid from past infections, or contributors to disease severity rather than the primary cause. Therefore, these findings highlight the complex etiology of pediatric ARI and underscore the need to delineate the specific pathogenic role of each detected microorganism and its potential interactions.

Previous studies suggest that co-detection of HPIV with other targeted respiratory agents may be associated with increased disease severity (24). While no significant differences in clinical presentation were reported between single and co-detection groups for HPIV-3 and HPIV-4 (12), the clinical impact of co-detection involving HPIV-1 and HPIV-2 remains unclear. In this study, the HPIV-1 mono-detection group was predominantly composed of infants aged ≤12 months (71.4%). In comparison, children in the co-detection group were older (median: 21.0 vs 6.0 months), had longer hospital stays, and incurred higher treatment costs. This pattern was further supported in stratified analyses, particularly among children co-detected with *Streptococcus pneumoniae*, rhinovirus, or *Mycoplasma pneumoniae*. Notably, total immunoglobulin levels (IgG, IgA, and IgM) were significantly higher in the co-detection group compared with the mono-detection group (all *P* < 0.05). Although immunoglobulin concentrations in children correlate positively with age (25), their elevation in this context may reflect an enhanced humoral immune response to complex or persistent microbial exposure (26, 27).

While previous studies on HPIV-1 have established its epidemiology, global burden, and role as a key pathogen in childhood lower respiratory infections—including potential extrapulmonary involvement (6, 28, 29)—detailed endoscopic, radiological, and complication data remain scarce. Our study compensates for this gap. Among the cases that underwent bronchoscopy, mucosal congestion and edema (91.8%) were the most prevalent findings, accompanied by a spectrum of characteristic secretions: white viscous secretions (75.4%), strip- or floc-like secretions in lavage fluid (65.6%), and wool-like secretions (36.1%). These frequencies provide a quantitative measure of airway lesion severity. On chest CT, the most prevalent abnormalities were patchy shadows (93.3%) and thickening of lung markings/bronchial walls (91.3%), while interstitial changes or unilateral consolidation were observed in 24.0% of cases. Together, these findings translate previously qualitative descriptions of inflammation into actionable, numerical indicators.

While extrapulmonary complications, such as cardiomyopathy, have been qualitatively reported (29), we systematically documented and determined the incidence of both respiratory (36.0%, predominantly pulmonary consolidation) and non-respiratory (40.2%, e.g., cardiomyopathy [18.2%] and hypokalemia [15.2%]) complications. Furthermore, we delineated the local disease burden, characterized by a median hospital stay of 7 days and a median treatment cost of 8,761.7 CNY, and observed a favorable prognosis with a 95.1% rate of improvement at discharge. These findings provide a quantitative complement to the existing epidemiological evidence (30).

Early identification of children at risk of severe disease is critical for improving clinical outcomes (31, 32). In this study, key early warning indicators for severe pneumonia among HPIV-1-positive children included significantly elevated inflammatory markers (PCT and ESR), prolonged fever, bilateral pulmonary involvement on imaging, and higher rates of complications and respiratory support needs. PCT is a well-established biomarker of systemic inflammation and bacterial involvement, whereas ESR reflects the intensity of inflammatory activity (33). The concurrent elevation of both markers in severe cases underscores a dysregulated inflammatory cascade that may drive disease progression. Together with clinical signs, such as prolonged fever and bilateral infiltrates, these laboratory parameters form a practical early-warning framework. This integrated approach provides clinicians with objective, measurable clues to promptly identify high-risk patients and guide targeted management—such as intensified anti-inflammatory treatment and close organ monitoring—to mitigate disease severity. Additionally, children with severe pneumonia more frequently required ICU care, experienced longer hospital stays, and incurred higher treatment costs, reflecting the substantial burden associated with disease progression. It should be noted that only 16 cases of severe pneumonia were included in this study. The limited sample size did not allow for multivariate regression analysis to assess the independent predictive value of these warning indicators. Future studies with larger cohorts are needed to validate their independence and prognostic utility.

This study has several limitations. First, as a single-center retrospective analysis, its findings require further validation in multicenter studies. Second, as a tNGS approach, the predefined primer panel may have missed rare or emerging pathogens not included in its detection spectrum. Third, nucleic acids detected by tNGS may in some cases reflect colonization, contamination, residual material from past infection, or latent infection rather than active disease. Although a thorough clinical and laboratory correlation was performed, this possibility cannot be fully excluded. Fourth, the pathogenic role of the multiple co-detected microbes and the mechanisms of interaction between HPIV-1 and other microbes remain unclear in our study population and warrant further investigation. Fifth, the limited number of HPIV-1 mono-detected cases (*n* = 7), particularly in the older age group, restricts the statistical power and reliability of comparisons between mono-detection and co-detection groups. Future prospective multicenter studies with standardized specimen collection protocols, larger sample sizes, and age-matched controls are warranted to validate our findings and clarify the clinical significance of polymicrobial respiratory infections.

Using tNGS, this study demonstrates that HPIV-1 detection in hospitalized children with ARI is characterized by a high rate of microbial co-detection, which is associated with greater disease severity. Elevated inflammatory markers (PCT and ESR), prolonged fever, complication occurrence, and need for respiratory support serve as key predictors of progression to severe pneumonia in these children. Together, these findings offer important clinical evidence to support the development of a predictive model for severe pneumonia in HPIV-1-positive children and provide valuable guidance for optimizing diagnostic and therapeutic strategies for HPIV-1-associated respiratory infections.

## Supplementary Material

Reviewer comments

## Data Availability

Data and materials may be made available upon written request to the corresponding author.

## SUPPLEMENTAL MATERIAL

Supplemental tables spectrum.00275-26-s0001.docx Tables S1 to S5.

OPEN PEER REVIEW reviewer-comments.pdf An accounting of the reviewer comments and feedback.

ASM does not own the copyrights to Supplemental Material that may be linked to, or accessed through, an article. The authors have granted ASM a non-exclusive, world-wide license to publish the Supplemental Material files. Please contact the corresponding author directly for reuse.
