# Supplementary material for: High prevalence and clinical impact of microbial co-detection in hospitalized children with human parainfluenza virus type 1
Source: Microbiol Spectr. 2026 Jun 16;14(7):e00275-26. doi: 10.1128/spectrum.00275-26 (PMC13340285; doi:10.1128/spectrum.00275-26)
Supplement: Supplemental tables — Tables S1 to S5. [file spectrum.00275-26-s0001.docx]

**Table S1**. 65 respiratory microorganisms (including 39 viruses, 18 bacteria, 4 fungi, and 4 atypical agents) detected by tNGS.

| **Microbial types (n)** | **Pathogens** |
| --- | --- |
| Viruses (n=39) | Influenza A virus, Influenza A virus (H1N1, 2009 pandemic H1N1, H3N2, H5N1, H7N9), Human adenovirus (groups B, C, E), Human parainfluenza virus (types 1, 2, 3, 4), Human rhinovirus (groups A, B, C), Human coronavirus (229E, HKU1, NL63, OC43), Human respiratory syncytial virus (A, B), Herpes simplex virus (1, 2), Human bocavirus (1, 4), Influenza B virus, Influenza C virus, Enterovirus, Enterovirus A71, Mumps virus, Parvovirus B-19, Rubella virus, Measles virus, Rotavirus, Human metapneumovirus, Varicella-zoster virus, Epstein-Barr virus, Cytomegalovirus. |
| Bacteria (n=18) | *Streptococcus pneumoniae, Serratia marcescens, Corynebacterium diphtheriae, Haemophilus influenzae, Streptococcus pyogenes, Klebsiella oxytoca, Moraxella catarrhalis, Legionella pneumophila, Stenotrophomonas maltophilia, Enterococcus faecalis, Enterococcus faecium, Bordetella pertussis, Mycobacterium tuberculosis* complex*, Nontuberculous mycobacteria, Klebsiella pneumoniae, Staphylococcus aureus, Acinetobacter baumannii, Pseudomonas aeruginosa.* |
| Fungi (n=4) | *Cryptococcus neoformans, Aspergillus fumigatus, Pneumocystis jirovecii, Candida albicans.* |
| Others (n=4) | *Chlamydophila pneumoniae, Mycoplasma pneumoniae, Chlamydia psittaci, Chlamydia trachomatis.* |

**Table S2**. 198 respiratory microorganisms (including 80 bacteria, 79 viruses, 32 fungi, and 7 atypical agents) detected by tNGS.

| **Microbial types (n)** | **Pathogens** |
| --- | --- |
| Viruses（n=79） | BK polyomavirus (Human polyomavirus type 1), Human adenovirus, Human adenovirus (types 1, 2, 3, 4, 5, 6, 7, 11, 14, 21, 34, 35, 55, 57; groups B, C, D), Human respiratory syncytial virus (A, B), Human rhinovirus, Human rhinovirus (groups A, B, C), Human herpesvirus (types 1 [Herpes simplex virus 1, HSV-1], 2 [Herpes simplex virus 2, HSV-2], 3 [Varicella-zoster virus, VZV], 4 [Epstein-Barr virus, EBV], 5 [Cytomegalovirus, CMV], 6, 7; 6A, 6B), Influenza A virus, Influenza A virus (H1N1, 2009 pandemic H1N1, H3N2, H5N1, H7N9), Influenza B virus, Influenza B virus (Victoria lineage, Yamagata lineage), Influenza C virus, JC polyomavirus (Human polyomavirus type 2), WU polyomavirus (Human polyomavirus type 4), Coxsackievirus (groups A2, A5, A6, A10, A16, B3), Echovirus (E18, E30), Enterovirus, Enterovirus (groups A, B, C, D; A71, D68), Human bocavirus (1, 2, 3, 4), Human coronavirus (229E, HKU1, NL63, OC43), Severe Acute Respiratory Syndrome Coronavirus 2, Human metapneumovirus, Human parainfluenza virus (types 1, 2, 3, 4), Human parvovirus B19, Measles virus, Mumps virus, Rubella virus. |
| Bacterium（n=80） | *Corynebacterium diphtheriae, Nocardia saintgeorgensis (Nocardia gelsenkirchenensis), Klebsiella aerogenes, Mycobacterium asiaticum, Nocardia farcinica, Klebsiella oxytoca, Mycobacterium avium, Nocardia nova, Klebsiella pneumoniae, Mycobacterium avium* complex (MAC), *Nocardia otitidiscaviarum, Klebsiella variicola, Mycobacterium occultum, Nocardia terpeneae, Legionella* spp*., Mycobacterium gordonae, Micromonospora parva, Legionella bozemanii, Mycobacterium intracellulare, Rhodococcus equi, Legionella pneumophila, Mycobacterium kansasii, Staphylococcus aureus, Legionella longbeachae, Mycobacterium malmoense, Streptococcus agalactiae, Legionella micdadei, nontuberculous mycobacteria* (NTM), *Streptococcus anginosus* group*, Moraxella catarrhalis, Mycobacterium scrofulaceum, Streptococcus intermedius, Acinetobacter baumannii, Mycobacterium schroeteri, Streptococcus pneumoniae, Acinetobacter johnsonii, Mycobacterium simiae, Streptococcus pyogenes, Acinetobacter ursingii, Mycobacterium szulgai, Tropheryma whipplei, Bacteroides fragilis, Mycobacterium tuberculosis* complex*, Peptostreptococcus anaerobius, Homburgeria, Mycobacterium ranarum, Burkholderia cepacia* complex*, Bordetella parapertussis, Mycobacterium abscessus, Burkholderia mallei, Bordetella pertussis, Mycobacterium chelonae-abscessus* complex*, Burkholderia pseudomallei, Brucella* spp.*, Mycobacterium chelonae, Burkholderia contaminans, Burkholderia cepacia nova, Mycobacterium fortuitum, Burkholderia multivorans, Burkholderia cepacia, Mycobacterium smegmatis, Elizabethkingia anophelis, Neisseria meningitidis, Nocardia* spp.*, Elizabethkingia meningoseptica, Pasteurella multocida, Nocardia abscessus, Enterobacter cloacae , Proteus mirabilis, Nocardia africana, Escherichia coli, Pseudomonas aeruginosa, Nocardia asteroides, Fusobacterium necrophorum, Serratia marcescens, Nocardia brasiliensis, Fusobacomplexcterium nucleatum, Stenotrophomonas maltophilia, Nocardia cavae, Haemophilus influenzae.* |
| Fungi (n=32) | *Candida glabrata, Fusarium* spp.*, Rhizomucor pusillus, Aspergillus flavus* complex*, Histoplasma capsulatum, Rhizopus* spp.*, Aspergillus fumigatus, Mucorales* spp.*, Rhizopus delemar, Aspergillus niger* complex*, Mucor plumbeus, Rhizopus microsporus, Aspergillus terreus* complex*, Mucor ramosissimus, Rhizopus oryzae, Candida albicans, Candida guilliermondii (Pichia guilliermondii), Cephalosporium* spp.*, Candida pseudoglabrata, Mucor irregularis, Cephalosporium acremonium, Candida parapsilosis, Mucor racemosus, Cephalosporium boydii, Candida tropicalis, Pichia kudriavzevii (Candida krusei), Talaromyces marneffei, Cryptococcus gattii, Pneumocystis jirovecii, Trichosporon asahii, Cryptococcus neoformans, Mucor rhizopus.* |
| Others (n=7) | *Chlamydophila pneumoniae, Mycoplasma pneumoniae, Coxiella burnetii, Chlamydia psittaci, Ureaplasma parvum, Chlamydia trachomatis, Ureaplasma urealyticum.* |

**Table S3.** Stratified analysis of human parainfluenza virus type 1 mono-detection versus co-detection with the top eight other microorganisms in hospitalized children with acute respiratory infection

| **Detected microbial patterns** | | **Age (months)** | | | **Length of hospitalization (days)** | | | **Treatment expense (CNY)** | | |
| --- | --- | --- | --- | --- | --- | --- | --- | --- | --- | --- |
| **Single** | **Multiple microbe**  **（At least include the following agens）** | **Mono-detection** | **Co-detection** | ***P*** value | **Mono-detection** | **Co-detection** | ***P*** value | **Mono-detection** | **Co-detection** | ***P*** value |
| HPIV-1 (n=7) | HPIV-1+*H. influenzae* (n=44) | 6.0  (1.1, 25.0) | 20.0  (10.3, 42.8) | **0.025** | 5.0  (4.0, 6.0) | 6.0  (5.0, 9.0) | 0.111 | 4142.3  (2773.6, 8408.5) | 7644.8  (5157.2, 10379.7) | 0.087 |
|  | HPIV-1+*S. pneumoniae* (n=44) | 6.0  (1.1, 25.0) | 21.5  (11.9, 47.3) | **0.023** | 5.0  (4.0, 6.0) | 7.0  (6.0, 9.8) | **0.013** | 4142.3  (2773.6, 8408.5) | 9765.1  (6275.7, 12523.3) | **0.013** |
|  | HPIV-1+HRV (n=41) | 6.0  (1.1, 25.0) | 33.0  (12.0, 50.0) | **0.016** | 5.0  (4.0, 6.00) | 7.0  (5.5, 9.0) | **0.028** | 4142.3  (2773.6, 8408.5) | 8746.9  (4925.1, 11921.5) | 0.070 |
|  | HPIV-1+*M. pneumoniae* (n=40) | 6.0  (1.1, 25.0) | 36.5  (16.0, 58.8) | **0.003** | 5.0  (4.0, 6.0) | 7.5  (6.0, 10.0) | **0.015** | 4142.3  (2773.6, 8408.5) | 9292.8  (8646.0, 11323.2) | **0.005** |
|  | HPIV-1+HCMV (n=38) | 6.0  (1.1, 25.0) | 10.4  (6.9, 17.5) | 0.234 | 5.0  (4.0, 6.0) | 8.5  (6.0, 11.0) | **0.005** | 4142.3  (2773.6, 8408.5) | 9327.4  (6071.9, 16890.4) | **0.012** |
|  | HPIV-1+*A. baumannii* (n=28) | 6.0  (1.1, 25.0) | 13.5  (8.5, 36.0) | 0.095 | 5.0  (4.0, 6.0) | 7.0  (6.0, 10.8) | **0.044** | 4142.3  (2773.6, 8408.5) | 7740.9  (4029.8, 11855.4) | 0.171 |
|  | HPIV-1+HAdV (n=25) | 6.0  (1.1, 25.0) | 15.0  (8.0, 31.5) | 0.092 | 5.0  (4.0, 6.0) | 7.0  (5.5, 9.0) | 0.054 | 4142.3  (2773.6, 8408.5) | 9656.8  (7923.5, 13569.6) | **0.018** |
|  | HPIV-1+*M. catarrhalis* (n=23) | 6.0  (1.1, 25.0) | 21.0  (11.0, 38.0) | 0.054 | 5.0  (4.0, 6.0) | 6.0  (5.0, 7.0) | 0.360 | 4142.3  (2773.6, 8408.5) | 7058.4  (3812.0, 8970.1) | 0.413 |

**Continue**

| **Detected microbial patterns** | | **WBC (×10^9^/L)** | | **NEU (%)** | | | | **LYM (%)** | | |
| --- | --- | --- | --- | --- | --- | --- | --- | --- | --- | --- |
| **Single** | **Multiple microbe**  **（At least include the following agens）** | **Mono-detection** | **Co-detection** | ***P*** value | **Mono-detection** | **Co-detection** | ***P*** value | **Mono-detection** | **Co-detection** | ***P*** value |
| HPIV-1 (n=7) | HPIV-1+*H. influenzae* (n=44) | 8.9±3.4 | 10.0±3.7 | 0.480 | 36.7±21.8 | 49.7±16.9 | 0.075 | 51.0±19.7 | 40.2±16.2 | 0.116 |
|  | HPIV-1+*S. pneumoniae* (n=44) | 8.9±3.4 | 9.5±2.7 | 0.578 | 36.7±21.8 | 49.9±16.3 | 0.063 | 51.0±19.7 | 39.5±14.6 | 0.072 |
|  | HPIV-1+HRV (n=41) | 8.9±3.4 | 9.1±3.7 | 0.865 | 36.7±21.8 | 45.3±17.1 | 0.241 | 51.0±19.7 | 43.7±15.4 | 0.272 |
|  | HPIV-1+*M. pneumoniae* (n=40) | 7.8 (6.3, 9.8) | 8.5 (6.1, 10.1) | 0.872 | 36.7±21.8 | 53.4±16.3 | **0.022** | 51.0±19.7 | 37.5±14.5 | **0.037** |
|  | HPIV-1+HCMV (n=38) | 8.9±3.4 | 10.1±3.4 | 0.378 | 36.7±21.8 | 41.9±17.2 | 0.487 | 51.0±19.7 | 47.3±16.8 | 0.601 |
|  | HPIV-1+*A. baumannii* (n=28) | 8.9±3.4 | 10.5±4.4 | 0.368 | 36.7±21.8 | 42.3±20.4 | 0.529 | 51.0±19.7 | 46.5±18.5 | 0.577 |
|  | HPIV-1+HAdV (n=25) | 8.9±3.4 | 11.3±3.7 | 0.139 | 36.7±21.8 | 48.4±18.3 | 0.160 | 51.0±19.7 | 42.2±17.3 | 0.255 |
|  | HPIV-1+*M. catarrhalis* (n=23) | 8.9±3.4 | 10.0±2.5 | 0.366 | 36.7±21.8 | 52.0±16.6 | 0.057 | 51.0±19.7 | 37.8±14.7 | 0.064 |

**Continue**

| **Detected microbial patterns** | | **IgG (g/L)** | | **IgA (g/L)** | | | | **IgM (g/L)** | | |
| --- | --- | --- | --- | --- | --- | --- | --- | --- | --- | --- |
| **Single** | **Multiple microbe**  **（At least include the following agens）** | **Mono-detection** | **Co-detection** | ***P*** value | **Mono-detection** | **Co-detection** | ***P*** value | **Mono-detection** | **Co-detection** | ***P*** value |
| HPIV-1 (n=7) | HPIV-1+*H. influenzae* (n=44) | 5.9±1.5 | 8.2±2.7 | **0.032** | 0.2 (0.2, 0.6) | 0.6 (0.3, 1.0) | 0.068 | 0.8±0.3 | 1.2±0.5 | **0.022** |
|  | HPIV-1+*S. pneumoniae* (n=44) | 5.9±1.5 | 8.1±2.4 | **0.024** | 0.4±0.2 | 0.8±0.5 | **0.035** | 0.8±0.3 | 1.3±0.5 | **0.004** |
|  | HPIV-1+HRV (n=41) | 6.4 (4.1, 6.7) | 8.5 (6.7, 10.6) | **0.006** | 0.2 (0.2, 0.6) | 0.7 (0.4, 1.4) | **0.049** | 0.8 (0.4, 1.1) | 1.2 (0.9, 1.8) | **0.006** |
|  | HPIV-1+*M. pneumoniae* (n=40) | 6.4 (4.1, 6.7) | 8.4 (7.5, 10.6) | **<0.001** | 0.2 (0.2, 0.6) | 0.8 (0.5, 1.5) | **0.008** | 0.8 (0.4, 1.1) | 1.4 (1.0, 1.9) | **0.002** |
|  | HPIV-1+HCMV (n=38) | 5.9±1.5 | 7.0±2.2 | 0.208 | 0.2 (0.2, 0.6) | 0.4 (0.1, 0.6) | 0.890 | 0.8±0.3 | 1.0±0.6 | 0.242 |
|  | HPIV-1+*A. baumannii* (n=28) | 5.9±1.5 | 8.0±3.3 | 0.103 | 0.2 (0.2, 0.6) | 0.4 (0.2, 0.9) | 0.300 | 0.8±0.3 | 1.2±0.7 | 0.119 |
|  | HPIV-1+HAdV (n=25) | 5.9±1.5 | 7.2±2.3 | 0.161 | 0.4±0.2 | 0.6±0.4 | 0.172 | 0.8 (0.4, 1.1) | 0.9 (0.7, 1.7) | 0.148 |
|  | HPIV-1+*M. catarrhalis* (n=23) | 5.9±1.5 | 8.2±2.6 | **0.030** | 0.4±0.2 | 0.8±0.5 | **0.031** | 0.8±0.3 | 1.2±0.5 | **0.036** |

**Continue**

| **Detected microbial patterns** | | **CYs-C (mg/L)** | | **CK (U/L)** | | | | **ALT (U/L)** | | |
| --- | --- | --- | --- | --- | --- | --- | --- | --- | --- | --- |
| **Single** | **Multiple microbe**  **（At least include the following agens）** | **Mono-detection** | **Co-detection** | ***P*** value | **Mono-detection** | **Co-detection** | ***P*** value | **Mono-detection** | **Co-detection** | ***P*** value |
| HPIV-1 (n=7) | HPIV-1+*H. influenzae* (n=44) | 1.2±0.3 | 0.9±0.2 | **0.027** | 66.0  (53.0, 86.0) | 92.0  (58.8, 139.0) | 0.060 | 22.0  (14.0, 59.0) | 15.0  (11.0, 25.8) | 0.104 |
|  | HPIV-1+*S. pneumoniae* (n=44) | 1.2±0.3 | 0.9±0.2 | **<0.001** | 66.0  (53.0, 86.0) | 92.5  (72.3, 119.3) | **0.037** | 22.0  (14.0, 59.0) | 14.0  (10.3, 19.8) | **0.027** |
|  | HPIV-1+HRV (n=41) | 1.1 (1.0, 1.6) | 0.9 (0.8, 1.0) | **0.013** | 66.0  (53.0, 86.0) | 98.0  (73.0, 132.0) | **0.022** | 22.0  (14.0, 59.0) | 17.0  (10.5, 23.5) | 0.147 |
|  | HPIV-1+*M. pneumoniae* (n=40) | 1.2±0.3 | 0.9±0.2 | **0.022** | 66.0  (53.0, 86.0) | 84.0  (58.3, 130.5) | 0.134 | 22.0  (14.0, 59.0) | 14.0  (10.0, 17.8) | **0.039** |
|  | HPIV-1+HCMV (n=38) | 1.2±0.3 | 1.0±0.2 | **0.030** | 66.0  (53.0, 86.0) | 92.0  (58.8, 139.0) | 0.163 | 22.0  (14.0, 59.0) | 17.0  (10.5, 23.5) | 0.591 |
|  | HPIV-1+*A. baumannii* (n=28) | 1.2±0.3 | 1.0±0.3 | 0.089 | 67.3±21.7 | 103.8±73.0 | 0.205 | 22.0  (14.0, 59.0) | 18.5  (12.3, 32.8) | 0.406 |
|  | HPIV-1+HAdV (n=25) | 1.2±0.3 | 0.9±0.2 | **0.036** | 67.3±21.7 | 102.5±45.3 | **0.009** | 22.0  (14.0, 59.0) | 17.0  (10.0, 28.0) | 0.346 |
|  | HPIV-1+*M. catarrhalis* (n=23) | 1.2±0.3 | 0.9±0.2 | **0.037** | 66.0  (53.0, 86.0) | 90.0  (72.0, 125.0) | **0.042** | 22.0  (14.0, 59.0) | 16.0  (13.0, 27.0) | 0.311 |

Note: HPIV-1, human parainfluenza virus type 1; WBC, white blood cell; NEU, neutrophil; LYM, lymphocyte; CYs-C, Cystatin C; CK-MB, creatine kinase-MB; ALT, alanine aminotransferase; *H. influenzae*, *Haemophilus influenzae*; *S. pneumoniae*, *Streptococcus pneumoniae*; HRV, human rhinovirus; *M. pneumoniae*, *Mycoplasma pneumoniae*; HCMV, human cytomegalovirus; *A. baumannii*, *Acinetobacter baumannii*; HAdV, human adenovirus; *M. catarrhalis*, *Moraxella catarrhalis*

**Table S4.** Specimen type distribution across age strata and detection rates of the top three co-detected pathogens by specimen type in human parainfluenza virus type 1-positive pediatric patients.

| **Specimen type distribution (%)** | **≤12 months (n=59)** | **>12 months (n=105)** | ***P* value** |  |
| --- | --- | --- | --- | --- |
| BALF | 26 (44.1%) | 66 (62.9%) | **0.020** |  |
| Throat swab | 29 (49.2%) | 37 (35.2%) | 0.081 |  |
| Sputum | 4 (6.8%) | 2 (1.9%) | 0.111 |  |
| **Detection rate of the top three co-detected pathogens (%)** | **BALF (n=92)** | **Throat swab (n=66)** | **Sputum (n=6)** | ***P* value** |
| *Haemophilus influenzae* | 23 (25.0%) | 20 (30.3%) | 1 (16.7%) | >0.05 |
| *Streptococcus pneumoniae* | 28 (30.4%) | 14 (21.2%) | 2 (33.3%) | >0.05 |
| Human rhinovirus | 22 (23.9%) | 18 (27.3%) | 1 (16.7%) | >0.05 |

Note: BALF, bronchoalveolar lavage fluid.

**Table S5.** Age-stratified subgroup analysis of clinical and laboratory features in human parainfluenza virus type 1-positive pediatric patients.

| **Characteristics** | **≤12 months  (Mono n=5 / Co n=54)** | ***P* value** | **>12 months  (Mono n=2 / Co n=98)** | ***P* value** |
| --- | --- | --- | --- | --- |
| **Quantitative data (median or mean)** |  |  |  |  |
| Age (months) | 2.0 / 8.0 | **0.023** | 36.0 / 37.0 | 0.092 |
| Cys-C (mg/L) | 1.5 / 1.1 | **0.048** | 0.9 / 0.9 | 0.690 |
| IgG (g/L) | 5.6 / 5.8 | 0.810 | 6.6 / 8.7 | **0.036** |
| IgA (g/L) | 0.2 / 0.3 | 0.505 | 0.7 / 0.8 | 0.453 |
| IgM (g/L) | 0.7 / 0.8 | 0.296 | 1.0 / 1.5 | 0.130 |
| Length of hospitalization (days) | 5.0 / 7.0 | **0.006** | 8.0 / 7.0 | 0.637 |
| Treatment expense (CNY) | 3358.0 / 9194.1 | **0.009** | 10248.6 / 8912.4 | 0.648 |

Note: Mono, human parainfluenza virus type 1 mono-detection; Co, human parainfluenza virus type 1 co-detection; CYs-C, Cystatin C.
