# Supplementary material for: High prevalence and clinical impact of microbial co-detection in hospitalized children with human parainfluenza virus type 1
Source: Microbiol Spectr. 2026 Jun 16;14(7):e00275-26. doi: 10.1128/spectrum.00275-26 (PMC13340285; doi:10.1128/spectrum.00275-26)
Supplement: Reviewer comments [file reviewer-comments.pdf]

AMERICAN SOCIETY FOR MICROBIOLOGY | Microbiology Spectrum

High Prevalence and Clinical Impact of Microbial Co-detection in Hospitalized Children with Human Parainfluenza Virus Type 1

Chunyun Fu, Xiaoying Chen, Lishai Mo, Junming Lu, Ya Huang, Jiangyang Zhao, Yuanyuan He, Juan Lin, Yanqing Tang, Shang Yi, Hao Wei, Xuehua Hu, and Jie Tan

Corresponding Author(s): Jie Tan, Maternal and Child Health Hospital of Guangxi Zhuang Autonomous Region

Review Timeline:

Submission Date:	January 22, 2026
Editorial Decision:	February 11, 2026
Revision Received:	March 27, 2026
Editorial Decision:	April 2, 2026
Revision Received:	April 3, 2026
Accepted:	April 7, 2026

Editor: Yuan Pin Hung

Reviewer(s): Disclosure of reviewer identity is with reference to reviewer comments included in decision letter(s). The following individuals involved in review of your submission have agreed to reveal their identity: Hussein Mohammed Al.Bayati, H.A.M (Reviewer #1)

Transaction Report:

(Note: With the exception of the correction of typographical or spelling errors that could be a source of ambiguity, letters and reports are not edited. The original formatting of letters and referee reports may not be reflected in this compilation.)

DOI: https://doi.org/10.1128/spectrum.00275-26

Re: Spectrum00275-26 (**High Prevalence and Clinical Impact of Microbial Co-detection in Hospitalized Children with Human Parainfluenza Virus Type 1**)

Dear Dr. Jie Tan:

Thank you for the privilege of reviewing your work. Below you will find my comments, instructions from the Spectrum editorial office, and the reviewer comments.

First, methodological bias in the tNGS approach is a concern. Since tNGS relies on a predefined primer panel, it inherently ignores pathogens outside that scope, potentially skewing the reported "complex microbial landscape." Furthermore, the PCR amplification step can introduce quantitative bias, meaning the "predominant patterns" observed might reflect amplification efficiency rather than true biological prevalence.
Second, the differentiation between colonization and infection remains insufficiently addressed. Common respiratory commensals like H. influenzae and S. pneumoniae often yield high read counts without causing disease. The study lacks a rigorous, bioinformatic-based threshold (e.g., relative abundance or viral load correlation) to distinguish true pathogens from transient flora.
Finally, the study establishes correlation, not causation. It remains unclear if co-detections drive severity or if severe HPIV-1 infection simply creates an opportunistic environment for secondary colonization.

Please return the manuscript within 60 days; if you cannot complete the modification within this time period, please contact me. If you do not wish to modify the manuscript and prefer to submit it to another journal, notify me immediately so that the manuscript may be formally withdrawn from consideration by Spectrum.

Revision Guidelines
To submit your modified manuscript, log into the submission site at https://spectrum.msubmit.net/cgi-bin/main.plex. Go to Author Tasks and click the appropriate manuscript title to begin. The information you entered when you first submitted the paper will be displayed; update this as necessary. Note the following requirements:

• Upload point-by-point responses to the issues raised by the reviewers in a file named "Response to Reviewers," NOT in your cover letter.
• Upload a compare copy of the manuscript (without figures) as a "Marked-Up Manuscript" file.
• Upload a clean .DOC/.DOCX version of the revised manuscript and remove the previous version.
• Each figure must be uploaded as a separate, editable, high-resolution file (TIFF or EPS preferred), and any multipanel figures must be assembled into one file.
• Any supplemental material intended for posting by ASM should be uploaded with their legends separate from the main manuscript. You can combine all supplemental material into one file (preferred) or split it into a maximum of 10 files with all associated legends included.

For complete guidelines on revision requirements, see our Submission and Review Process webpage. Submission of a paper that does not conform to guidelines may delay acceptance of your manuscript.

Data availability: ASM policy requires that data be available to the public upon online posting of the article, so please verify all links to sequence records, if present, and make sure that each number retrieves the full record of the data. If a new accession number is not linked or a link is broken, provide Spectrum production staff with the correct URL for the record. If the accession numbers for new data are not publicly accessible before the expected online posting of the article, publication may be delayed; please contact production staff (Spectrum@asmusa.org) immediately with the expected release date.

Publication Fees: For information on publication fees and which article types are subject to charges, visit our website. If your manuscript is accepted for publication and any fees apply, you will be contacted separately about payment during the production process; please follow the instructions in that e-mail. Arrangements for payment must be made before your article is published.

ASM Membership: Corresponding authors may join or renew ASM membership to obtain discounts on publication fees. Need to upgrade your membership level? Please contact Customer Service at Service@asmusa.org.

The ASM Journals program strives for constant improvement in our submission and publication process. Please tell us how we can improve your experience by taking this quick Author Survey.

Thank you for submitting your paper to Spectrum.

Sincerely,
Yuan Pin Hung
Editor

Reviewer #1 (Comments for the Author):

Hello dear
I want to express my admiration in your work , just required some re writing of discussion with recent references and supported with your opinion dear

Reviewer #2 (Comments for the Author):

Considering that bacterial infections detected at higher quantities than viral infection may it caused by instability of viral RNA genome in comparison to bacterial DNA genome?
Also some of the co detected bacteria might colonize the airways looking for copy number of these bacteria might be beneficial.
Also as looking for host RNA something like GAPDH or other highly express housekeeping RNA in the airway to assure correct sample storage and preparation is recommended.

Reviewer #3 (Comments for the Author):

See attached file.

Reviewer #4 (Comments for the Author):

This study describes the analysis of human parainfluenza virus type 1 infection in a large cohort (~9,000) of hospitalized children, utilizing next-generation sequencing to reveal the prevalence of HPIV-1 and the presence of viral and bacterial co-infections. The manuscript is well-written and despite the reliance on NGS, which will detect commensal pathogens, the authors combine their findings with in depth clinical analysis that is well laid out and comprehensive to ensure only patients with active infections are identified. The findings are clear and well-presented and offer a new tool for clinicians to understand the prevalence and symptoms of HPIV-1 infection in different age groups. Important findings include the age group difference in prevalence of infection and co-infection and the difference in hospitalization rates. Overall, the manuscript provides a robust and methodologically modern look at pediatric acute respiratory infection due to HPIV-1.

This manuscript is very interesting and useful for the field. My only minor critique is to remove statements on antibody titers increasing with infection as it does not appear these were tested in a way where antigen-specificity is known against HPIV-1.

Reviewer #5 (Comments for the Author):

In this study, the authors characterized the epidemiology, microbial co-detection profiles, and associated clinical features of HPIV-1 in hospitalized children with acute respiratory infections using targeted next-generation sequencing. This is a well-conducted and clearly written study, and the conclusions are supported by the data. I have only a few minor comments for the authors' consideration.

1.
The abstract is clear but includes many numbers, especially in the Results section. The authors may consider reducing the number of details and focusing on the main findings to improve readability.

2.
A brief statement describing HPIV as an RNA virus in the family Paramyxoviridae may help non-specialist readers.

High Prevalence and Clinical Impact of Microbial Co-detection in Hospitalized Children with Human Parainfluenza Virus Type 1

Chunyun Fu[1#], Xiaoying Chen[1#], Lishai Mo[1#], Junming Lu[1], Ya Huang[2], Jiangyang Zhao[1], Yuanyuan He[2], Juan Lin[2], Yanqing Tang[3], Shang Yi[3], Hao Wei[3], Xuehua Hu[1]*, Jie Tan[2]*

[1]Medical Science Laboratory, Children's Hospital, Maternal and Child Health Hospital of Guangxi Zhuang Autonomous Region, Nanning 530003, People's Republic of China

[2]Department of Pediatric Respiratory Medicine, Children's Hospital, Maternal and Child Health Hospital of Guangxi Zhuang Autonomous Region, Nanning 530003, People's Republic of China

[3]Department of Genetic Metabolism, Children's Hospital, Maternal and Child Health Hospital of Guangxi Zhuang Autonomous Region, Nanning 530003, People's Republic of China

[#] Chunyun Fu, Xiaoying Chen, and Lishai Mo contribute equally to this work.

*Address correspondences to:

Jie Tan, E-mail: tanjie273@163.com; Xuehua Hu, E-mail: huxuehua16@sina.com

No.59 Xiangzhu Road

Nanning 530003

Guangxi Zhuang Autonomous Region, P. R. China

Fax: (+86-771)-2860 792

ABSTRACT

Objective: To investigate the detection rate, co-detection patterns, and clinical outcomes of human parainfluenza virus type 1 (HPIV-1) in hospitalized children with acute respiratory infections (ARI), and identify indicators for severe pneumonia.

Methods: 10,153 children with ARI initially screened by targeted next-generation sequencing (tNGS), 9,394 met the inclusion criteria. Clinical features were compared between HPIV-1 mono-detection and co-detection groups, as well as between patients with severe and non-severe pneumonia.

Results: HPIV-1 was detected in 1.75% (164/9,394) of children, with 43.29% of positive cases aged 6 months–2 years. Microbial co-detection occurred in 95.73% (157/164) of HPIV-1-positive children, predominantly involving both viruses and bacteria (42.68%). The most common co-detected microorganisms were *Haemophilus influenzae*, *Streptococcus pneumoniae*, Rhinovirus, and *Mycoplasma pneumoniae* (each >25.00%). The mono-detection group predominantly consisted of infants aged ≤12 months (71.43%). In contrast, the co-detection group was older (median 21.0 vs. 6.0 months), had higher immunoglobulin levels, longer hospitalizations, and higher costs (P<0.05). Stratified analysis confirmed these trends. Among HPIV-1-positive cases, respiratory complications, non-respiratory complications, and clinical improvement/discharge rates were 35.98%, 40.24%, and 95.12%, respectively. Compared to non-severe cases, patients with severe pneumonia had significantly elevated inflammatory markers, longer fever duration, higher complications and ICU admission rates, greater respiratory support, longer hospitalizations, and higher costs (P<0.05).

Conclusion: HPIV-1-positive pediatric ARI patients have a high co-detection rate, with HPIV-1-virus-bacteria co-detection being the predominant pattern. Co-detection is linked to greater disease severity compared to mono-detection. Key indicators for severe pneumonia

include elevated inflammatory markers, prolonged fever, complications, and respiratory support needs.

IMPORTANCE

Human parainfluenza virus type 1 (HPIV-1) is an underrecognized contributor to pediatric acute respiratory infections (ARI), yet its clinical impact remains unclear, particularly regarding co-detection with other microorganisms. Based on targeted next-generation sequencing (tNGS), this study demonstrates that HPIV-1 detection in hospitalized children with ARI is characterized by a high rate of microbial co-detection, which is associated with greater disease severity. Elevated inflammatory markers (PCT and ESR), prolonged fever, complication occurrence, and need for respiratory support serve as key predictors of progression to severe pneumonia in these children. These findings offer important clinical evidence to support the development of a predictive model for severe pneumonia in HPIV-1-positive children and provide valuable guidance for optimizing diagnostic and therapeutic strategies for HPIV-1-associated respiratory infections.

Keywords: human parainfluenza virus type 1; acute respiratory infection; tNGS; co-detection; children

INTRODUCTION

Acute respiratory infections (ARI) are one of the leading causes of hospitalization and mortality in children under 5 years of age worldwide[1]. Viruses are the most common cause of pediatric respiratory infections[2]. According to the first global burden estimates for acute lower respiratory infections in children, human parainfluenza virus (HPIV) infections account for approximately 13% of all pediatric acute lower respiratory infection cases and are associated with 4% to 14% of related hospitalizations[1], contributing substantially to healthcare burden. HPIV comprises four serotypes (HPIV-1 to HPIV-4), with HPIV-1 being

associated with the largest and most well-documented outbreaks[3, 4]. In young children, HPIV-1 is particularly known to cause severe clinical manifestations such as laryngotracheobronchitis, bronchiolitis, and pneumonia, and may also compromise immune function[3, 5, 6, 7], thereby posing significant challenges in clinical management. As a result, HPIV-1 has been recognized as an important pathogen in children hospitalized with ARI.

Despite the established role of HPIV-1 as a significant pediatric pathogen, critical gaps remain in two key areas. First, technical limitations persist. Most studies have relied on quantitative real-time PCR [8, 9]. Though specific, this method may fail to detect novel strains or cases with low viral loads due to its targeted nature, potentially leading to an underestimation of the detection rate. Second, the prevalence, patterns, and clinical impact of HPIV-1 co-detection with other agents remain poorly characterized[8, 9].

This study utilized targeted next-generation sequencing (tNGS)—a method chosen for its sensitivity and broad pathogen coverage[10, 11] —to screen over 10,000 pediatric ARI inpatients. It aimed to investigate the epidemiology, co-detection patterns, and clinical features of HPIV-1-positive children. Comparisons were made between the mono-detection and microbial co-detection groups, and between severe and non-severe pneumonia cases. The results are expected to inform the clinical management of HPIV-1-associated disease in children.

MATERIALS AND METHODS

Study population

This retrospective study analyzed 10,153 pediatric patients hospitalized with ARI at the Maternal and Child Health Hospital of Guangxi Zhuang Autonomous Region between April 2021 and December 2024. Clinical information and laboratory data were collected for all included cases. ARI is defined as an acute clinical condition resulting from various pathogens,

characterized by the sudden onset of respiratory symptoms and typically lasting no more than 21 days. Common clinical manifestations include cough, sputum production, shortness of breath, sore throat, and rhinorrhea. The spectrum of ARI encompasses acute upper respiratory tract infections, acute bronchitis, and community-acquired pneumonia.

Definition of severe and non-severe pneumonia

Severe pneumonia was defined as radiologically confirmed pneumonia meeting at least one of the following major criteria: (1) general danger signs (e.g., impaired consciousness, convulsions, inability to feed, dehydration, or severe vomiting); (2) respiratory distress or hypoxemia (e.g., central cyanosis, $SpO_2 \leq 92\%$ on room air, grunting, severe tachypnea, or chest wall indrawing); (3) respiratory failure necessitating non-invasive or invasive mechanical ventilation; (4) radiological evidence of complications (e.g., multi-lobar involvement, pleural effusion, or necrotizing pneumonia); (5) systemic complications (e.g., hemodynamic instability, shock, heart failure, acute kidney injury, or other organ dysfunction).

For this study, organ dysfunction was defined as meeting one or more of the following criteria: (1) cardiovascular dysfunction: requiring vasoactive agents or presenting with septic shock; (2) neurological dysfunction: glasgow Coma Score ≤11 or a rapid decline of ≥3 points; (3) renal dysfunction: serum creatinine ≥2 times the age-specific upper limit of normal or requiring renal replacement therapy; (4) hepatic dysfunction: hyperbilirubinemia, coagulopathy, or transaminase elevation >5 times the upper limit of normal; (5) hematological dysfunction: disseminated intravascular coagulation or severe thrombocytopenia. Notably, isolated mild transaminase elevations (≤5 times the upper limit of normal) without other hepatic impairment were excluded from this classification.

Non-severe pneumonia was diagnosed in children with compatible clinical symptoms and radiological evidence of pneumonia, provided that none of the severe pneumonia criteria were

met. These patients typically presented with fever, cough, and tachypnea, but without signs of severe respiratory distress, hypoxemia, or major systemic complications.

Inclusion and exclusion criteria

Included cases had to meet all the following criteria: aged ≤14 years, hospitalized due to an acute respiratory tract infection, and having undergone tNGS testing with available results. Cases were excluded for any of the following reasons: incomplete essential clinical or laboratory data (e.g., missing demographics, tNGS reports, or clinical diagnosis records); a confirmed non-infectious etiology for respiratory symptoms (e.g., allergies, asthma, or environmental irritants); or a diagnosis of hospital-acquired infection.

Targeted next-generation sequencing

A total of 10,153 clinical specimens—including 4,578 bronchoalveolar lavage fluid samples, 4,648 throat swabs, and 927 sputum samples—were collected under standardized clinical protocols[12]. Total nucleic acid extraction was performed using the MagPure Pathogen DNA/RNA Kit (R6672-01B, Magen, Guangzhou, China). Subsequent library preparation for targeted next-generation sequencing (tNGS) was performed with the Respiration100™ multiplex PCR system (KingCreate, Guangzhou, China) to detect common respiratory pathogens, including HPIV-1. It should be noted that the manufacturer's panel was updated during the study period, leading to a significant expansion of the detection spectrum from an initial 65 to a final 198 targeted agents (**Supplementary Tables 1 and 2**). The major analytical platforms utilized for tNGS data processing and analysis were the DRAGEN Bio-IT Platform, Galaxy, and CLC Genomics Workbench. A comprehensive description of the tNGS methodology and workflow has been published previously[13]. The interpretation of

targeted sequencing results relied on two key metrics: amplicon coverage and normalized read count. Detection of microorganisms was defined according to the following criteria: (i) For bacteria (excluding the *Mycobacterium tuberculosis* complex), fungi, and atypical pathogens: amplicon coverage ≥ 50% and normalized read count ≥ 10; (ii) For viruses: amplicon coverage ≥ 50% and normalized read count ≥ 3, or a normalized read count ≥ 10; (iii) For the *Mycobacterium tuberculosis* complex: a normalized read count ≥ 1 was considered indicative of detection.

Definition of infection versus colonization

Subsequently, two experienced clinicians independently evaluated the clinical data to determine the relevance of potential pathogens to the ARI and overall clinical presentation. Their comprehensive assessment incorporated the patient's medical history, clinical symptoms, results of routine microbiological tests, inflammatory markers, epidemiological features, imaging findings, tNGS results, and other laboratory data. In cases of disagreement, a senior consultant was consulted to reach a consensus. To ensure diagnostic accuracy and systematically differentiate true pathogenic infection from colonization, this evaluation was guided by a multidimensional framework that assessed four clinical domains: (1) host factors (e.g., immunosuppression due to neutropenia, HIV, transplantation, or prematurity); (2) microbiological evidence (e.g., elevated pathogen burden via tNGS, low viral PCR Ct values, invasive forms on microscopy, or positive cultures from sterile sites); (3) clinical and radiographic features (e.g., new or progressive infiltrates, ground-glass opacities, viral patterns on imaging, or elevated markers such as procalcitonin, C-reactive protein, galactomannan, or virus-specific antibodies); and (4) therapeutic response (e.g., clinical improvement within 72 hours of targeted therapy or confirmed virological clearance).

Statistical analysis

Statistical analyses were performed using IBM SPSS Statistics version 26.0. Measurement data conforming to a normal distribution with homogeneity of variance are presented as the mean ± standard deviation ($\bar{x} \pm s$), and comparisons between two groups were conducted using the independent samples t-test. Data not following a normal distribution are expressed as the median (interquartile range), and the Mann-Whitney U test was used for comparisons between two groups of non-parametric data. Categorical data are presented as frequency (percentage, %), and intergroup comparisons were made using the chi-square (χ^2) test. The goodness-of-fit chi-square test analyzed distribution differences. P<0.05 was considered statistically significant.

RESULTS

Detection rate of HPIV-1

Among the 10,153 hospitalized children with ARI and tested tNGS, a total of 9,394 cases met the inclusion criteria for the study **(Figure 1)**. Among them, 226 cases had negative tNGS results, and 9,168 cases had positive tNGS results, with a positive rate of 97.59% (9,168/9,394). 164 cases were detected with HPIV-1, with a detection rate of 1.75% (164/9,394). In terms of specimen types, the detection rate of HPIV-1 was the highest in bronchoalveolar lavage fluid (2.09%, 92/4,403), followed by throat swabs (1.60%, 66/4,120) and sputum (0.69%, 6/871).

Demographics of HPIV-1-positive children with ARI

The 164 HPIV-1-positive children showed an uneven age distribution (P<0.001), with cases concentrated in the 0.5–1 year (20.73%) and 1–2 years (22.56%) age groups; the median age was 20.0 months (IQR: 10.0–45.8 months). Males were significantly more prevalent than females (60.98% vs 39.02%; P<0.05). The distribution across hospital admission departments was also significantly different (P<0.001). The majority were admitted to the Pediatric

Respiratory Department (58.54%), followed by the Pediatric Cardiology Department (15.24%); the remaining cases were distributed among other departments. Detailed demographic information is presented in **Table 1**.

Epidemiological and detection characteristics of HPIV-1 in hospitalized children with ARI

From April 2021 to December 2024, 164 children hospitalized with ARI were positive for HPIV-1. Cases exhibited a distinct seasonal distribution, predominantly in autumn and winter, with peaks in November 2022, October 2023, and January 2024 **(Figure 2A)**. Mono-detection was uncommon (n=7, 4.27%). Most cases (n=157, 95.73%) involved co-detection **(Figures 1, 2B)**, most frequently with two (n=41, 25.00%) or three (n=37, 22.56%) other microorganisms **(Figure 2B)**. The predominant compositional pattern was HPIV-1-virus-bacteria co-detection (n=70, 42.68%), followed by HPIV-1-virus-bacteria-atypical agent co-detection (n=21, 12.80%) (**Figure 2C**). tNGS identified 53 distinct microorganisms co-detected with HPIV-1, comprising 18 viruses, 24 bacteria, 7 fungi, and 4 atypical agents (**Figure 2D**). The primary age distribution of the top five co-detected agents was as follows: *Haemophilus influenzae* and *Streptococcus pneumoniae* (both most common in children aged 6 months–2 years and 3–5 years), Human rhinovirus (3–5 years), *Mycoplasma pneumoniae* (1–2 years and 3–5 years), and Human cytomegalovirus (6 months–2 years) **(Figure 2E)**. In contrast, children with HPIV-1 mono-detection were predominantly aged ≤12 months (5 out of 7, 71.43%) (**Figures 2B, C**).

Features of HPIV-1 mono-detection and co-detection

Clinical and laboratory features of the HPIV-1 mono-detection (n=7) and co-detection (n=157) groups are presented in **Table 2**. Children with co-detection were significantly older (median 21.0 vs. 6.0 months, P=0.019). They also exhibited significantly lower cystatin C (0.90 vs. 1.11 mg/L, P=0.015) and higher levels of IgG (8.02 vs. 6.44 g/L), IgA (0.62 vs. 0.23 g/L), and

IgM (1.20 vs. 0.80 g/L) (all *P*<0.05). Clinically, the co-detection group had longer hospitalizations (7.0 vs. 5.0 days, *P*=0.033) and higher treatment costs (8912.35 vs. 4142.27 CNY, *P*=0.030). A stratified analysis based on the top eight co-detected microorganisms yielded results largely consistent with the overall co-detection group (**Supplementary Table 3**).

Bronchoscopy and computed tomography (CT) findings

Of the 164 HPIV-1-positive children, 61 (37.20%) underwent bronchoscopy (**Figure 3A**). The main abnormalities observed were mucosal congestion and edema (n=56, 91.80%), white viscous mucosal secretions (n=46, 75.41%), strip- or floc-like secretions in lavage fluid (n=40, 65.57%), and wool-like secretions (n=22, 36.07%).

Chest CT was performed in 104 children (63.41%) (**Figure 3B**). The predominant abnormal findings included patchy opacities (n=97, 93.27%), thickening of lung markings and bronchial walls (n=95, 91.35%), linear opacities (n=25, 24.04%), unilateral consolidation (n=25, 24.04%), and bilateral consolidation (n=18, 17.31%).

Complications and respiratory support

Of the 164 children with HPIV-1-associated ARI, 59 (35.98%) developed respiratory complications, primarily pulmonary consolidation (n=37, 62.71%), hypoxia (n=10, 16.95%), and respiratory failure (n=10, 16.95%) **(Figure 3C)**. Non-respiratory complications occurred in 66 children (40.24%), mainly including cardiomyopathy (n=12, 18.18%), intestinal dysfunction (n=10, 15.15%), hypokalemia (n=10, 15.15%), and neutropenia (n=8, 12.12%) (**Figure 3C**).

Respiratory support was required in 28 cases (17.07%). Non-invasive respiratory support was provided to 15 children (53.57% of those requiring support), comprising supplemental oxygen therapy (n=10, 66.76%), non-invasive ventilation (n=3, 20.00%), and continuous positive airway pressure (CPAP, n=2, 13.33%). Invasive respiratory support was administered to 13

children (46.4%), all via endotracheal intubation or tracheostomy coupled with invasive mechanical ventilation **(Figure 3D)**.

Prognosis

Of the 164 children with HPIV-1-associated ARI, the primary clinical diagnosis was bronchitis (43.90%, 72/164), followed by bilateral pneumonia (33.54%, 55/164) and unilateral pneumonia (7.93%, 13/164). Severe pneumonia was diagnosed in 16 children (9.76%), 11 of whom required ICU admission. The median hospital stay was 7.0 days (IQR: 5.0–10.0 days), with a median treatment cost of 8,761.71 CNY (IQR: 5,403.51–12,094.52 CNY). Following standard symptomatic treatment, 156 children (95.12%) improved and were discharged. The remaining 8 cases comprised 2 discharges against medical advice (due to family refusal of treatment) and 6 voluntary discharges (attributed to perceived insufficient efficacy or financial constraints).

Comparison of clinical features between severe and non-severe pneumonia in children with HPIV-1

Clinical features differed significantly between children with severe and non-severe HPIV-1-associated pneumonia **(Table 3)**. Compared with the non-severe group, the severe group had higher levels of procalcitonin (PCT) (0.48 vs. 0.09 ng/mL, $P<0.001$) and erythrocyte sedimentation rate (ESR) (38.00 vs. 20.00 mm/h, $P=0.007$), longer duration of fever (7.0 vs. 2.0 days, $P<0.001$) and hospital stay (17.5 vs. 6.0 days, $P<0.001$), and higher treatment costs (57,725.49 vs. 8,357.11 CNY, $P<0.001$). The severe group had significantly higher rates of bilateral pneumonia (68.75% vs. 29.73%, $P=0.002$), ICU admission (68.75% vs. 0.00%, $P<0.001$), respiratory complications (93.75% vs. 29.73%, $P<0.001$), and non-respiratory complications (81.25% vs. 35.81%, $P=0.001$). Additionally, the severe group more frequently required respiratory support (87.50% vs. 9.46%, $P<0.001$) and invasive respiratory support (68.75% vs. 1.35%, $P<0.001$), and had a lower rate of favorable discharge

(81.25% vs. 96.62%, $P<0.001$). Although statistically significant differences were noted in measures such as white blood cell count (WBC) and neutrophil percentage (NEU%), the median/mean values for all these parameters fell within normal reference ranges.

DISCUSSION

HPIV-1 is an important etiological agent of ARI in children. While previous studies have described the epidemiology and genetic features of HPIV-1 in pediatric populations[6, 14], none have systematically analyzed its epidemiology, microbial co-detection profiles, and associated clinical characteristics using tNGS in a large cohort of hospitalized children with ARI. To address this gap, our study enrolled 10,153 hospitalized ARI children tested by tNGS (9,394 meeting inclusion criteria) and demonstrated a high rate of microbial co-detection with HPIV-1. Furthermore, we identified early-warning indicators for severe pneumonia in children with HPIV-1-associated illness, offering clinically useful guidance.

Compared with conventional methods, tNGS enables more comprehensive microbial profiling by specifically enriching target nucleic acids through techniques such as primer amplification or hybridization capture[11]. In this study, tNGS detection revealed an overall HPIV-1 positivity rate of 1.75% among 9,394 hospitalized children with ARI, with the highest rate (2.09%) observed in bronchoalveolar lavage fluid. Affected children were predominantly male (60.98%) and aged 6 months to 2 years, with a higher frequency of detection in autumn and winter—findings that align with previously reported epidemiological trends for HPIV-1[3, 15]. A key finding was the high rate of microbial co-detection with HPIV-1: up to 95.73% of HPIV-1-positive children had other respiratory microorganisms detected concurrently, most commonly in an HPIV-1-virus-bacteria pattern. This rate substantially exceeds those reported in studies using traditional detection methods[16, 17]; for example, one multiplex PCR-based study reported a co-detection rate of 24.8% for multiple respiratory pathogens[18], underscoring the capacity of tNGS to better reveal the complex microbial landscape in

respiratory illnesses. Among co-detected microorganisms, *Haemophilus influenzae*, *Streptococcus pneumoniae*, Rhinovirus, and *Mycoplasma pneumoniae* were the most frequent (each >25.00%). It is important to note that not all co-detected microorganisms are necessarily causative pathogens; some may represent colonization, residual nucleic acid from past infections, or contributors to disease severity rather than the primary cause. Therefore, these findings highlight the complex etiology of pediatric ARI and underscore the need to delineate the specific pathogenic role of each detected microorganism and its potential interactions.

Previous studies suggest that co-detection of HPIV with other microorganisms may be associated with increased disease severity[18]. While no significant differences in clinical presentation were reported between single and co-detection groups for HPIV-3 and HPIV-4[19], the clinical impact of co-detection involving HPIV-1 and HPIV-2 remains unclear. In this study, the HPIV-1 mono-detection group was predominantly composed of infants aged ≤12 months (71.43%). In comparison, children in the co-detection group were older (median: 21.0 vs. 6.0 months), had longer hospital stays, and incurred higher treatment costs. This pattern was further supported in stratified analyses, particularly among children co-detected with *Streptococcus pneumoniae*, rhinovirus, or *Mycoplasma pneumoniae*. Notably, immunoglobulin levels (IgG, IgA, and IgM) were generally higher in the co-detection group. Although immunoglobulin concentrations in children correlate positively with age[20], their elevation in this context may reflect an enhanced humoral immune response to complex or persistent microbial exposure[21, 22].

While previous studies on HPIV-1 have established its epidemiology, global burden, and role as a key pathogen in childhood lower respiratory infections—including potential extrapulmonary involvement [6, 23, 24]—detailed endoscopic, radiological, and complication data remain scarce. Our study compensates for this gap. Among the cases that underwent bronchoscopy, mucosal congestion and edema (91.80%) were the most prevalent findings,

accompanied by a spectrum of characteristic secretions: white viscous secretions (75.41%), strip- or floc-like secretions in lavage fluid (65.57%), and wool-like secretions (36.07%). These frequencies provide a quantitative measure of airway lesion severity. On chest CT, the most prevalent abnormalities were patchy shadows (93.27%) and thickening of lung markings/bronchial walls (91.35%), while interstitial changes or unilateral consolidation were observed in 24.04% of cases. Together, these findings translate previously qualitative descriptions of inflammation into actionable, numerical indicators.

While extrapulmonary complications such as cardiomyopathy have been qualitatively reported [24], we systematically documented and determined the incidence of both respiratory (35.98%, predominantly pulmonary consolidation) and non-respiratory (40.24%, e.g., cardiomyopathy [18.18%] and hypokalemia [15.15%]) complications. Furthermore, we delineated the local disease burden, characterized by a median hospital stay of 7 days and a median treatment cost of 8,761.71 CNY, and observed a favorable prognosis with a 95.12% rate of improvement at discharge. These findings provide a quantitative complement to the existing epidemiological evidence [25].

Early identification of children at risk of severe disease is critical for improving clinical outcomes[26, 27]. In this study, key early warning indicators for severe pneumonia among HPIV-1-positive children included significantly elevated inflammatory markers (PCT and ESR), prolonged fever, bilateral pulmonary involvement on imaging, and higher rates of complications and respiratory support needs. PCT is a well-established biomarker of systemic inflammation and bacterial involvement, whereas ESR reflects the intensity of inflammatory activity[28]. The concurrent elevation of both markers in severe cases underscores a dysregulated inflammatory cascade that may drive disease progression. Together with clinical signs such as prolonged fever and bilateral infiltrates, these laboratory parameters form a practical early-warning framework. This integrated approach provides clinicians with

objective, measurable clues to promptly identify high-risk patients and guide targeted management—such as intensified anti-inflammatory treatment and close organ monitoring—to mitigate disease severity. Additionally, children with severe pneumonia more frequently required ICU care, experienced longer hospital stays, and incurred higher treatment costs, reflecting the substantial burden associated with disease progression. It should be noted that only 16 cases of severe pneumonia were included in this study. The limited sample size did not allow for multivariate regression analysis to assess the independent predictive value of these warning indicators. Future studies with larger cohorts are needed to validate their independence and prognostic utility.

This study has several limitations. First, as a single-center retrospective analysis, its findings require further validation in multicenter studies. Second, nucleic acids detected by tNGS may in some cases reflect colonization, contamination, residual material from past infection, or latent infection rather than active disease. Although a thorough clinical and laboratory correlation was performed, this possibility cannot be fully excluded. Third, the pathogenic role of the multiple co-detected microorganisms and the mechanisms of interaction between HPIV-1 and other microbes remain unclear in our study population and warrant further investigation.

Using tNGS, this study demonstrates that HPIV-1 detection in hospitalized children with ARI is characterized by a high rate of microbial co-detection, which is associated with greater disease severity. Elevated inflammatory markers (PCT and ESR), prolonged fever, complication occurrence, and need for respiratory support serve as key predictors of progression to severe pneumonia in these children. Together, these findings offer important clinical evidence to support the development of a predictive model for severe pneumonia in HPIV-1-positive children and provide valuable guidance for optimizing diagnostic and therapeutic strategies for HPIV-1-associated respiratory infections.

AUTHOR CONTRIBUTORS

Chunyun Fu, Xiaoying Chen, and Jie Tan drafted the original manuscript. Xuehua Hu and Lishai Mo critically revised the manuscript. All the authors contributed to the data collection and statistical analysis, and approved the final manuscript.

FUNDING

This study was supported by the Guangxi Medical and Health Appropriate Technology Development and Promotion Application Project (S2024082) and the Guangxi Health Commission self-financing projects (Z-A20220257).

DISCLOSURE STATEMENT

No potential conflict of interest was reported by the author(s).

HUMAN ETHICS AND CONSENT TO PARTICIPATE

The study was approved by the Medical Ethics Committee of Maternal and Child Health Hospital of Guangxi Zhuang Autonomous Region. Informed consent from the patients was waived due to the retrospective nature of the study.

AVAILABILITY OF DATA AND MATERIALS

Data and materials may be made available upon written request to the corresponding author.

FIGURE LEGENDS

Figure 1. Study flow diagram: Cohort selection, tNGS testing, and HPIV-1 analysis in children with acute respiratory infections. ARI, acute respiratory infection; tNGS, targeted next-generation sequencing; HPIV-1, human parainfluenza virus 1; BALF, bronchoalveolar lavage fluid.

Figure 2. Epidemiological and co-detection profiles of HPIV-1 in pediatric acute respiratory infection.

(A) Monthly HPIV-1 detections by tNGS (April 2021–December 2024).

(B) Distribution of the number of co-detected microorganisms by age group.

(C) Distribution of co-detection patterns by age group.

(D) Top 10 detected viruses, bacteria, fungi, and atypical agents.

(E) Top 21 co-detected microorganisms by age group.

HPIV-1, human parainfluenza virus type 1; MO, microorganisms; M, months; Y, years.

Figure 3. Clinical examinations, complications, and respiratory support in children with HPIV-1 acute respiratory infection.

(A) Bronchoscopic findings (n=61).

(B) Chest computed tomography findings (n=104).

(C) Spectrum of respiratory (n=59) and non-respiratory (n=66) complications.

(D) Utilization of non-invasive and invasive respiratory support (n=28).

HPIV-1, human parainfluenza virus type 1; COT, central oxygen therapy; MV, mechanical ventilation; CPAP, continuous positive airway pressure; MV (via ETT/Trach), mechanical ventilation via endotracheal tube/tracheostomy.

REFERENCES

1. Wang X, Li Y, Deloria-Knoll M, Madhi SA, Cohen C, Arguelles VL, et al. Global burden of acute lower respiratory infection associated with human parainfluenza virus in children younger than 5 years for 2018: a systematic review and meta-analysis. The Lancet Global health. 2021;9(8):e1077-e1087. DOI: 10.1016/s2214-109x(21)00218-7.
2. van Woensel JB, van Aalderen WM, Kimpen JL. Viral lower respiratory tract infection in infants and young children. BMJ (Clinical research ed). 2003;327(7405):36-40. DOI: 10.1136/bmj.327.7405.36.
3. Hall CB. Respiratory syncytial virus and parainfluenza virus. The New England journal of medicine. 2001;344(25):1917-28. DOI: 10.1056/nejm200106213442507.
4. Vainionpää R, Hyypiä T. Biology of parainfluenza viruses. Clinical microbiology reviews. 1994;7(2):265-75. DOI: 10.1128/cmr.7.2.265.
5. Henrickson KJ. Parainfluenza viruses. Clinical microbiology reviews. 2003;16(2):242-64. DOI: 10.1128/cmr.16.2.242-264.2003.
6. Guo YJ, Sun J, Li YL, Lai QR, Li L, Zhou HY, et al. Epidemiology and genetic characterization of human parainfluenza virus-1 infection in pediatric patients from Hangzhou China, 2021-2022. Virology journal. 2024;21(1):206. DOI: 10.1186/s12985-024-02479-8.
7. Adderson E, Allison KJ, Branum K, Sealy RE, Jones BG, Surman SL, et al. Intranasal Sendai Virus Vaccination of Seropositive Children 1 to 2 Years of Age in a Phase I Clinical Trial Boosts Immune Responses Toward Human Parainfluenza Virus Type 1. Vaccines. 2025;13(4). DOI: 10.3390/vaccines13040430.
8. Howard LM, Edwards KM, Zhu Y, Williams DJ, Self WH, Jain S, et al. Parainfluenza Virus Types 1-3 Infections Among Children and Adults Hospitalized With Community-acquired Pneumonia. Clinical infectious diseases : an official publication

of the Infectious Diseases Society of America. 2021;73(11):e4433-e4443. DOI: 10.1093/cid/ciaa973.

9. Han JY, Suh W, Han SB. Seasonal epidemiological and clinical characteristics of pediatric patients with human parainfluenza virus infection by serotype: a retrospective study. Virology journal. 2022;19(1):141. DOI: 10.1186/s12985-022-01875-2.

10. Chen Q, Yi J, Liu Y, Yang C, Sun Y, Du J, et al. Clinical diagnostic value of targeted next-generation sequencing for infectious diseases (Review). Molecular medicine reports. 2024;30(3). DOI: 10.3892/mmr.2024.13277.

11. Healthcare SoCMoCIEaPAfMa. [Expert consensus on the application and practice of targeted next-generation sequencing in infectious diseases]. Zhonghua yi xue za zhi. 2024;104(48):4375-4383. DOI: 10.3760/cma.j.cn112137-20240927-02208.

12. Pediatric Bronchoscopy Collaborative Group TSGoRD, The Society of Pediatrics, Chinese Medical Association. [Guide to pediatric bronchoscopy (2009 edition)]. Zhonghua er ke za zhi = Chinese journal of pediatrics. 2009;47(10):740-4.

13. Deng Z, Li C, Wang Y, Wu F, Liang C, Deng W, et al. Targeted next-generation sequencing for pulmonary infection diagnosis in patients unsuitable for bronchoalveolar lavage. Frontiers in medicine. 2023;10:1321515. DOI: 10.3389/fmed.2023.1321515.

14. Marx A, Török TJ, Holman RC, Clarke MJ, Anderson LJ. Pediatric hospitalizations for croup (laryngotracheobronchitis): biennial increases associated with human parainfluenza virus 1 epidemics. The Journal of infectious diseases. 1997;176(6):1423-7. DOI: 10.1086/514137.

15. DeGroote NP, Haynes AK, Taylor C, Killerby ME, Dahl RM, Mustaquim D, et al. Human parainfluenza virus circulation, United States, 2011-2019. Journal of clinical

virology : the official publication of the Pan American Society for Clinical Virology. 2020;124:104261. DOI: 10.1016/j.jcv.2020.104261.

16. Chen ZR, Ji W, Wang YQ, Yan YD, Shao XJ, Zhang XL, et al. Etiology of acute bronchiolitis and the relationship with meteorological conditions in hospitalized infants in China. Journal of the Formosan Medical Association = Taiwan yi zhi. 2014;113(7):463-9. DOI: 10.1016/j.jfma.2012.12.013.

17. Yasir M, Al-Sharif HA, Al-Subhi T, Sindi AA, Bokhary DH, El-Daly MM, et al. Analysis of the nasopharyngeal microbiome and respiratory pathogens in COVID-19 patients from Saudi Arabia. Journal of infection and public health. 2023;16(5):680-688. DOI: 10.1016/j.jiph.2023.03.001.

18. Zhong P, Zhang H, Chen X, Lv F. Clinical characteristics of the lower respiratory tract infection caused by a single infection or coinfection of the human parainfluenza virus in children. Journal of medical virology. 2019;91(9):1625-1632. DOI: 10.1002/jmv.25499.

19. Xiao NG, Duan ZJ, Xie ZP, Zhong LL, Zeng SZ, Huang H, et al. Human parainfluenza virus types 1-4 in hospitalized children with acute lower respiratory infections in China. Journal of medical virology. 2016;88(12):2085-2091. DOI: 10.1002/jmv.24580.

20. Bayram RO, Özdemir H, Emsen A, Türk Dağı H, Artaç H. Reference ranges for serum immunoglobulin (IgG, IgA, and IgM) and IgG subclass levels in healthy children. Turkish journal of medical sciences. 2019;49(2):497-505. DOI: 10.3906/sag-1807-282.

21. Sebina I, Pepper M. Humoral immune responses to infection: common mechanisms and unique strategies to combat pathogen immune evasion tactics. Current opinion in immunology. 2018;51:46-54. DOI: 10.1016/j.coi.2018.02.001.

22. Kräutler NJ, Yermanos A, Pedrioli A, Welten SPM, Lorgé D, Greczmiel U, et al. Quantitative and Qualitative Analysis of Humoral Immunity Reveals Continued and Personalized Evolution in Chronic Viral Infection. Cell reports. 2020;30(4):997-1012.e6. DOI: 10.1016/j.celrep.2019.12.088.

23. Zhu Z, Zhang Y, Mao NY. Human parainfluenza virus: an important but overlooked respiratory pathogen. World journal of pediatrics : WJP. 2025;21(3):220-222. DOI: 10.1007/s12519-025-00888-5.

24. Farahmand M, Shatizadeh Malekshahi S, Jabbari MR, Shayestehpour M. The landscape of extrapulmonary manifestations of human parainfluenza viruses: A systematic narrative review. Microbiology and immunology. 2021;65(1):1-9. DOI: 10.1111/1348-0421.12865.

25. King C, Colbourn T. Global human parainfluenza virus estimates for action on childhood pneumonia. The Lancet Global health. 2021;9(8):e1033-e1034. DOI: 10.1016/s2214-109x(21)00259-x.

26. Qin YJ, Yang YX, Li JX, Guan J. [Risk factors for hypoxemia in children with severe Mycoplasma pneumoniae pneumonia]. Zhongguo dang dai er ke za zhi = Chinese journal of contemporary pediatrics. 2025;27(2):192-198. DOI: 10.7499/j.issn.1008-8830.2408094.

27. Almirall J, Bolíbar I, Toran P, Pera G, Boquet X, Balanzó X, et al. Contribution of C-reactive protein to the diagnosis and assessment of severity of community-acquired pneumonia. Chest. 2004;125(4):1335-42. DOI: 10.1378/chest.125.4.1335.

28. Van den Bruel A, Thompson MJ, Haj-Hassan T, Stevens R, Moll H, Lakhanpaul M, et al. Diagnostic value of laboratory tests in identifying serious infections in febrile children: systematic review. BMJ (Clinical research ed). 2011;342:d3082. DOI: 10.1136/bmj.d3082.

Table 1. Demographic characteristics of children with HPIV-1-associated acute respiratory infection

**Characteristics**	**Patients infected with HPIV-1 (n=164)**	***P* value**
**Age**		
Median age (months), (IQR)	20.00 (10.00, 45.75)	
≤3 months, n (%)	12 (7.32%)	
3-6 months, n (%)	13 (7.39%)	
6-12 months, n (%)	34 (20.73%)	
1-2 years, n (%)	37 (22.56%)	**<0.001**
2-3 years, n (%)	15 (9.15%)	
3-5 years, n (%)	32 (19.51%)	
5-12 years, n (%)	21 (12.80%)	
**Gender**		
Male, n (%)	100 (60.98%)	**<0.01**
Female, n (%)	64 (39.02%)	
**Department**		
Pediatric Respiratory Medicine	96 (58.54%)	
Pediatric Cardiology	25 (15.24%)	
Pediatric Intensive Care Unit	11 (6.71%)	
Pediatric Infectious Diseases	8 (4.88%)	
Pediatric Nephrology	7 (4.27%)	**<0.001**
Pediatric Gastroenterology	7 (4.27%)	
Pediatric Neurology	6 (3.66%)	
Pediatric General Surgery	2 (1.22%)	
Neonatology	1 (0.61%)	
Pediatric Otolaryngology	1 (0.61%)	

Note: HPIV-1, human parainfluenza virus type 1; IQR, interquartile range.

Table 2. Comparison of laboratory parameters and clinical features between children with HPIV-1 mono-detection and those with microbial co-detection.

**Characteristics**	**HPIV-1 Mono-detection (n=7)**	**HPIV-1 Co-detection (n=157)**	***P* value**
**Quantitative data [Median (IQR)] or [Mean±SD]**			
Age (months)	6.00 (1.13, 25.00)	21.00 (10.85, 46.50)	**0.019**
RBC ($\times10^{12}$/L)	4.30 (3.80, 4.70)	4.60 (4.30, 4.95)	0.180
WBC ($\times10^{9}$/L)	7.80 (6.30, 9.80)	9.10 (6.70, 11.55)	0.566
NEU (%)	36.70±21.76	47.03±18.93	0.162
LYM (%)	51.00±19.72	42.68±17.50	0.222
Mon (%)	9.00 (8.50, 13.30)	8.00 (5.55, 10.75)	0.219
Eos (%)	1.50 (0.10, 3.50)	0.70 (0.10, 1.55)	0.509
Baso (%)	0.20 (0.00, 0.20)	0.20 (0.10, 0.35)	0.106
PLT ($\times10^{9}$/L)	397.00 (307.00, 498.00)	338.00 (265.50, 448.00)	0.251
CRP (mg/L)	1.62 (0.50, 22.55)	2.76 (0.56, 7.77)	0.667
PCT (ng/mL)	0.10 (0.09, 0.26)	0.10 (0.05, 0.23)	0.471
AST (U/L)	35.00 (31.00, 105.00)	37.00 (31.00, 47.50)	0.684
ALT (U/L)	22.00 (14.00, 59.00)	16.00 (12.00, 26.00)	0.160
ESR (mm/h)	17.00 (15.00, 26.00)	20.00 (13.00, 33.50)	0.720
D-dimer (ng/mL FEU)	449.00 (360.00, 1270.00)	430.00 (292.00, 750.00)	0.489
LDH (U/L)	323.00 (293.00, 424.00)	313.00 (276.00, 351.50)	0.251
URE (mmol/L)	20.00 (15.00, 30.00)	26.00 (20.00, 32.00)	0.153
CYs-C (mg/L)	1.11 (0.96, 1.56)	0.90 (0.80, 1.06)	**0.015**
CK (U/L)	66.00 (53.00, 86.00)	88.00 (60.50, 125.00)	0.078
CK-MB (U/L)	27.00 (23.00, 36.00)	26.00 (20.00, 31.50)	0.336
IgG (g/L)	6.44 (4.12, 6.70)	8.02 (6.38, 9.60)	**0.014**
IgA (g/L)	0.23 (0.19, 0.62)	0.62 (0.32, 1.04)	**0.047**
IgM (g/L)	0.80 (0.39, 1.09)	1.20 (0.83, 1.66)	**0.014**
Duration of fever (days)	2.00 (0.00, 2.00)	2.00 (0.00, 3.00)	0.334
Length of hospitalization (days)	5.00 (4.00, 6.00)	7.00 (5.00, 10.00)	**0.033**
Treatment expense (CNY)	4142.27 (2773.61, 8408.51)	8912.35 (5754.67, 12277.33)	**0.030**
**Categorical data [n (%)]**			
Gender (Male)	3 (42.86%)	97 (61.78%)	0.543
Fever	4 (57.14%)	113 (71.97%)	0.673
Intensive care unit (ICU) admission	0 (0.00%)	11 (7.01%)	1.000
Bronchitis	3 (42.86%)	69 (43.95%)	1.000
Bilateral pneumonia	2 (28.57%)	53 (33.76%)	1.000
Unilateral pneumonia	1 (14.29%)	12 (7.64%)	1.000
Bronchopneumonia	0 (0.00%)	5 (3.18%)	1.000
Severe pneumonia	0 (0.00%)	16 (10.19%)	1.000
Respiratory complications	1 (14.29%)	58 (36.94%)	0.412
Non-respiratory complications	2 (28.57%)	64 (40.76%)	0.803
Respiratory support	0 (0.00%)	28 (17.83%)	0.604
Invasive respiratory support	0 (0.00%)	13 (8.28%)	1.000
Discharged with improvement	7 (100.00%)	149 (94.90%)	1.000

Note: HPIV-1, human parainfluenza virus type 1; IQR, interquartile range; SD, standard deviation; RBC, red blood cell; WBC, white blood cell; NEU, neutrophil; LYM, lymphocyte; Mon, monocyte; Eos, eosinophil; Baso, basophil; PLT, platelet; CRP, C-reactive protein; PCT, procalcitonin; AST, aspartate aminotransferase; ALT, alanine aminotransferase; ESR, erythrocyte sedimentation rate; LDH, lactate dehydrogenase; URE, urea; CYs-C, Cystatin C; CK, creatine kinase; CK-MB, creatine kinase-MB.

Table 3. Clinical characteristics of children with HPIV-1-associated pneumonia: Severe versus non-severe cases.

Characteristics	Severe pneumonia (n=16)	Non-severe pneumonia (n=148)	*P* value
**Quantitative data [Median (IQR)] or [Mean±SD]**			
Age (months)	20.50 (8.28, 75.00)	20.00 (10.00, 45.00)	0.874
RBC ($\times 10^{12}$/L)	4.15 (3.53, 5.33)	4.60 (4.30, 4.90)	0.204
WBC ($\times 10^{9}$/L)	9.70 (8.35 14.60)	8.90 (6.43, 11.30)	**0.048**
NEU (%)	62.77±18.77	44.84±18.35	**<0.001**
LYM (%)	29.84±17.45	44.46±17.08	**0.001**
Mon (%)	5.55 (4.55, 9.10)	8.35 (6.35, 10.95)	**0.024**
Eos (%)	0.10 (0.00, 0.90)	0.70 (0.20, 1.75)	**0.002**
Baso (%)	0.20 (0.10, 0.30)	0.20 (0.10, 0.40)	0.384
PLT ($\times 10^{9}$/L)	360.50 (267.75, 431.50)	341.00 (267.50, 450.50)	0.794
CRP (mg/L)	4.34 (1.23, 28.16)	2.70 (0.50, 7.17)	0.151
PCT (ng/mL)	0.48 (0.14, 3.53)	0.09 (0.05, 0.22)	**<0.001**
AST (U/L)	39.50 (28.25, 53.00)	37.00 (31.00, 47.75)	0.784
ALT (U/L)	19.00 (13.25, 47.75)	16.50 (12.00, 26.00)	0.284
ESR (mm/h)	38.00 (15.50, 55.00)	20.00 (13.00, 29.00)	**0.007**
D-dimer (ng/mL FEU)	785.00 (270.00, 11307.50)	430.00 (302.50, 672.50)	0.172
LDH (U/L)	344.00 (268.50, 381.00)	308.50 (277.25, 348.00)	0.186
URE (mmol/L)	27.00 (21.99, 41.00)	25.00 (19.21, 32.00)	0.286
CYs-C (mg/L)	1.02 (0.78, 1.45)	0.90 (0.81, 1.06)	0.168
CK (U/L)	60.00 (45.00, 97.50)	89.00 (64.00, 125.00)	**0.031**
CK-MB (U/L)	25.00 (17.50, 30.00)	26.00 (21.00, 32.00)	0.293
IgG (g/L)	7.08 (4.91, 10.33)	8.00 (6.45, 9.42)	0.490
IgA (g/L)	0.75 (0.24, 1.76)	0.61 (0.31, 1.00)	0.610
IgM (g/L)	1.22 (0.85, 1.98)	1.19 (0.80, 1.64)	0.624
Duration of fever (days)	7.00 (1.50, 18.00)	2.00 (0.00, 3.00)	**<0.001**
Length of hospitalization (days)	17.50 (9.50, 24.50)	6.00 (5.00, 9.00)	**<0.001**
Treatment expense (CNY)	57725.49 (18936.84, 98507.85)	8357.11 (5117.94, 10688.89)	**<0.001**
**Categorical data [n (%)]**			
Gender (Male)	11 (68.75%)	89 (60.14%)	0.502
Fever	14 (87.50%)	103 (69.59%)	0.225
Patients with HPIV-1 co-infection	0 (0.00%)	7 (4.73%)	1.000
Bronchitis	5 (31.25%)	67 (45.27%)	0.283
Bilateral pneumonia	11 (68.75%)	44 (29.73%)	**0.002**
Unilateral pneumonia	0 (0.00%)	13 (8.78%)	0.368
Bronchopneumonia	0 (0.00%)	5 (3.39%)	1.000
Intensive care unit (ICU) admission	11 (68.75%)	0 (0.00%)	**<0.001**
Respiratory complications	15 (93.75%)	44 (29.73%)	**<0.001**
Non-respiratory complications	13 (81.25%)	53 (35.81%)	**0.001**
Respiratory support	14 (87.50%)	14 (9.46%)	**<0.001**
Invasive respiratory support	11 (68.75%)	2 (1.35%)	**<0.001**
Discharged with improvement	13 (81.25%)	143 (96.62%)	**0.036**

Note: HPIV-1, human parainfluenza virus type 1; IQR, interquartile range; SD, standard deviation; RBC, red blood cell; WBC, white blood cell; NEU, neutrophil; LYM, lymphocyte; Mon, monocyte; Eos, eosinophil; Baso, basophil; PLT, platelet; CRP, C-reactive protein; PCT, procalcitonin; AST, aspartate aminotransferase; ALT, alanine aminotransferase; ESR, erythrocyte sedimentation rate; LDH, lactate dehydrogenase; URE, urea; CYs-C, Cystatin C; CK, creatine kinase; CK-MB, creatine kinase-MB.

Journal: Microbiology Spectrum

Type of manuscript: Research Article

Manuscript ID: Spectrum00275-26

Title: High Prevalence and Clinical Impact of Microbial Co-detection in Hospitalized Children with Human Parainfluenza Virus Type 1

Summary: This manuscript describes a large retrospective study of clinical and laboratory records obtained from pediatric patients hospitalized with ARI at a single hospital in Nanning, Guangxi, China, from April 2021 to Dec 2024. Case enrollment required available test results from a large panel of respiratory pathogens using next generation sequencing of preamplified targets (tNGS). Analysis of 10,153 pediatric patients yielded 9,394 children that met specified selection criteria. Of these, HPIV-1 was detected in 164 (1.75%) by tNGS. Demographic, epidemiological and clinical data of HPIV-1 patients were analyzed and those with discrete and multiple pathogen infections compared. The authors concluded that these findings will advance efforts to develop algorithms that predict clinical progression of HPIV-1 ARI. The study findings are confirmatory rather than novel and it remains a question how these data can be used to build accurate predictive models.

Major concerns:

1) **Detection methodology.** The tNGS detection methodology used in this study appears flawed. No SARS-CoV-2 was included in the panel(s), despite it being an important cause of ARI prevalent during the study period. Also, it is inconceivable that HPIV-1 would be the most prevalent pathogen detected in this demographic, suggesting tNGS targeting bias or HPIV-1 target contamination. Moreover, the authors report that an "updated" manufacturers respiratory pathogen panel was introduced "during the study period". All samples must be tested by one or the other panel, which is to say that the data from different panel tests cannot be combined unless confirmed that identical test results would be obtained.
2) S**tudy design.** The retrospective study design using a single time-point specimen collection/testing of non-standard specimen types (where specimen type may correlate with patient age and therefore influence specific pathogen types detected) without age-matched controls compromises clinical outcome prediction. The few patients with mono-HPIV-1-detections (7) further complicates comparisons with co-detection data (Lines 209-218, Table 2). This is a particular problem where age distribution bias between the two groups could explain differences in the measured variables (children under one year of age would be expected to have fewer codetections because respiratory pathogen burden is directly correlated with age).
3) **Infection vs. colonization**. The contribution of "potential pathogens to the ARI and overall clinical presentation" (Lines 148-163) cannot be accurately established for most of these pathogens by the proposed methodology. Establishing a pathogen's role in

disease requires additional information; e.g., pathogen load, measured changes in pathogen load, serology to establish acute infection or pathology of affected organs/tissues. The attributable contribution to disease with multiple pathogen detections would be even more difficult to establish.

Other comments:

1) Why were older children excluded from the study (enrolled aged ≤14 years)? The internationally accepted definition of a child is generally any person under the age of 18.
2) Do not present data out to 3 decimal places, as this is unsupportable accuracy (example, Table 2).
3) Be consistent with italicizing genus/species names (e.g., Supplementary Tables 1 &2; Figure 2). Try to consolidate names where appropriate (e.g., parainfluenza virus types 1,2,3).
4) Figure 1. Exclusion should be ">14 years".
5) Line 186. "Males were significantly more prevalent than females …". This finding for HPIV-1 patients cannot be interpreted without knowing what the proportion of males and females was in the overall study sample.
6) Lines 198, 201, 296. The term "microorganisms" suggests all respiratory tract flora when only those associated with ARI were tested (respiratory pathogens).
7) Lines 281-282. Peak circulation of HPIV-1 occurs during odd-numbered years in temperate regions which was not demonstrated in this study.

Mar 27, 2026

Editorial Board

Microbiology Spectrum

Dear Editor,

We greatly appreciate the valuable comments provided by the reviewers and Editor, which have significantly contributed to improving our manuscript **"High Prevalence and Clinical Impact of Microbial Co-detection in Hospitalized Children with Human Parainfluenza Virus Type 1"** (Spectrum00275-26). We have revised the manuscript accordingly and **highlighted in red in the updated version**. Our responses to the Editor's comments are outlined below.

Editor's comments:

1. First, methodological bias in the tNGS approach is a concern. Since tNGS relies on a predefined primer panel, it inherently ignores pathogens outside that scope, potentially skewing the reported "complex microbial landscape." Furthermore, the PCR amplification step can introduce quantitative bias, meaning the "predominant patterns" observed might reflect amplification efficiency rather than true biological prevalence.

Response: Thank you for your valuable comments. We address your concerns as follows:

Regarding the first concern: Limitations of the predefined primer panel

We agree that tNGS, relying on a predefined primer panel, may inherently miss pathogens outside its scope. During this study, the Respiration100™ panel was expanded from 65 to 198 target agents (Table S1 and S2), covering common and relatively rare respiratory pathogens. However, we acknowledge the theoretical possibility of missing rare or emerging pathogens. We have also included this point as a limitation in our Discussion section **(Manuscript, Lines 362–364, Page 16)**.

Regarding the second concern: Quantitative bias introduced by PCR amplification

You raise an important point about PCR amplification efficiency differences, which is a critical factor in tNGS. To minimize its impact on our conclusions, we implemented the following measures in our experimental design and data analysis:

1) Rigorous quality control system: Each batch included exogenous internal controls and negative/positive controls to monitor amplification efficiency. Only data passing quality control were included, effectively controlling systematic bias.
2) Quantitative interpretation criteria: Pathogen detection required dual thresholds—amplicon coverage ≥50% and normalized read count ≥3 (or ≥10) for viruses, and ≥10 for bacteria/fungi. These thresholds were specifically designed to exclude low-burden colonization. Pathogens with artificially inflated reads due to amplification bias but insufficient coverage were not classified as predominant.
3) Comprehensive clinical adjudication: A key strength of our study is that the reported "predominant patterns" were not solely based on tNGS quantitation. Two experienced clinicians independently integrated host factors, radiographic features, laboratory findings, and therapeutic response to confirm true infections. This multidisciplinary approach effectively mitigates biases from any single technical metric.
4) Consistency of detection protocols: Although the panel was updated during the study, the detection protocols, primer/probe sequences, and interpretation thresholds for key pathogens (including HPIV-1) remained identical, ensuring data comparability across different periods.

2. Second, the differentiation between colonization and infection remains insufficiently addressed. Common respiratory commensals like H. influenzae and S. pneumoniae often yield high read counts without causing disease. The study lacks a rigorous, bioinformatic-based threshold (e.g., relative abundance or viral load correlation) to distinguish true pathogens from transient flora.

Response: Thank you for raising this important point. We fully agree that distinguishing colonization from true infection—particularly for common respiratory commensals such as Haemophilus influenzae and Streptococcus pneumoniae—is a

critical challenge in interpreting tNGS results.

First, while we acknowledge that a purely bioinformatic threshold (e.g., relative abundance or viral load correlation) can be useful, we believe that such thresholds alone are insufficient to definitively differentiate colonization from infection. This is because the pathogen load required to cause disease varies significantly depending on host immune status, specimen type, and clinical context. A fixed threshold may misclassify low-load true infections in immunocompromised patients or high-load colonization in healthy carriers.

Second, to address this challenge, our study employed a comprehensive multidimensional clinical adjudication framework, rather than relying solely on a bioinformatic cutoff. As described in the Methods section, two experienced clinicians independently evaluated each case by integrating:

- Microbiological evidence: including tNGS read counts, amplicon coverage, and—for key commensals—their relative abundance within the total microbial community;
- Specimen type: detection in bronchoalveolar lavage fluid (BALF) was weighted more heavily than in throat swabs;
- Clinical and radiographic findings: new infiltrates, ground-glass opacities, fever, purulent sputum, and elevated inflammatory markers (e.g., PCT, CRP);
- Host factors: immunocompromised status (e.g., neutropenia, transplantation) lowered the threshold for considering a commensal as pathogenic;
- Therapeutic response: clinical improvement within 72 hours of targeted therapy served as retrospective confirmation of clinical relevance.

Third, for the specific commensals mentioned (H. influenzae, S. pneumoniae), we applied stricter criteria when they were detected in throat swabs. In such cases, they were classified as pathogens only when: (a) there was strong clinical and radiographic evidence of infection, (b) they were present at high abundance or predominance relative to other bacteria, and (c) alternative pathogens were reasonably excluded. Detections lacking this clinical correlation were considered colonization and excluded from all analyses.

We thank the editor for this valuable comment, which has helped us strengthen the clarity of our methodology.

3. Finally, the study establishes correlation, not causation. It remains unclear if co-detections drive severity or if severe HPIV-1 infection simply creates an opportunistic environment for secondary colonization.

Response: Thank you for this insightful comment. We fully agree that our study, as a retrospective observational analysis, demonstrates correlation rather than causation regarding the relationship between pathogen co-detections and disease severity in HPIV-1-infected children.

First, we acknowledge that the observed associations between co-detection and more severe clinical outcomes could be interpreted in two ways: (1) polymicrobial infections directly contribute to increased disease severity; or (2) severe HPIV-1 infection disrupts respiratory mucosal barriers and immune defenses, creating an opportunistic environment that facilitates secondary colonization or detection of commensal organisms. Our current study design cannot definitively distinguish between these mechanisms.

Second, despite this limitation, we believe our findings provide valuable epidemiological evidence that co-detections are clinically relevant. As shown in Supplementary Table 3, compared to children with HPIV-1 mono-detection, those with co-detections — particularly involving Streptococcus pneumoniae, Mycoplasma pneumoniae, or Haemophilus influenzae—exhibited significantly longer hospital stays, higher treatment costs, and more pronounced elevations in inflammatory markers. While these associations do not prove causality, they suggest that co-detection identifies a subgroup of children at higher risk for complicated clinical courses, which may inform clinical management and risk stratification.

Third, we have now explicitly acknowledged this limitation in the Discussion section of our revised manuscript. We note that further studies—particularly prospective cohort studies with serial sampling, animal models, or mechanistic in vitro experiments—are warranted to elucidate whether co-detections are drivers of disease severity or

markers of host susceptibility.

We thank the editor for highlighting this important distinction, which has helped us more precisely frame the scope and clinical implications of our work.

Reviewer 1:

1. Hello dear

I want to express my admiration in your work, just required some re writing of discussion with recent references and supported with your opinion dear.

Response: Thank you very much for your kind words and positive assessment of our work. We also appreciate your constructive suggestion to revise the Discussion section by incorporating recent references and our own interpretive perspectives.

In response, we have updated the Discussion with two high-quality, recently published studies to strengthen the academic rigor of our analysis and to support our interpretation of key findings. Specifically, we have cited impactful articles from Nature Communications and Euro Surveillance to contextualize the altered epidemiological pattern of HPIV-1 observed in our study, which we attribute to the disruption of respiratory virus circulation during the COVID-19 pandemic [20,21] **(Manuscript, Lines 292–295, Page 13)**.

We believe these additions have improved the depth and evidence base of the manuscript. The references are as follows:

20. Zhao C, Zhang T, Guo L, Sun S, Miao Y, Yung CF, et al. Characterising the asynchronous resurgence of common respiratory viruses following the COVID-19 pandemic. Nat Commun. 2025;16(1):1610. DOI: 10.1038/s41467-025-56776-z.

21. Laird TS, Hamilton M, William N, Karanwal S, Marsh K, Evans J. Trends in human parainfluenza virus in Scotland before and after the peak of the COVID-19 pandemic, January 2017 to October 2023. Euro Surveill. 2025;30(2). DOI: 10.2807/1560-7917.Es.2025.30.2.2400147.

Reviewer 2:

1. Considering that bacterial infections detected at higher quantities than viral infection may it caused by instability of viral RNA genome in comparison to bacterial DNA genome?

Also some of the co detected bacteria might colonize the airways looking for copy number of these bacteria might be beneficial. Also as looking for host RNA something like GAPDH or other highly express housekeeping RNA in the airway to assure correct sample storage and preparation is recommended.

Response: Thank you for raising these important technical considerations regarding the tNGS methodology. We address your concerns as follows:

(1) Regarding the stability difference between viral RNA and bacterial DNA:

We fully agree that RNA viruses are generally less stable than DNA bacteria, and theoretical differential degradation could potentially affect detection accuracy. To minimize this potential bias, we implemented the following measures in our experimental workflow:

- Immediate low-temperature storage after collection: All clinical specimens were placed in refrigerators immediately after collection to minimize the risk of nucleic acid degradation at room temperature.
- Regular experimental frequency: We maintained an experimental frequency of once every 1-2 days, ensuring a short interval between specimen collection and testing to avoid nucleic acid loss that may occur with prolonged storage.
- Reverse transcription step: Our tNGS workflow includes a reverse transcription step that converts viral RNA into complementary DNA (cDNA), which is more stable and enables comparable amplification with bacterial DNA.
- Multiplex PCR optimization: The Respiration100™ panel is specifically optimized for simultaneous detection of RNA and DNA targets, with primer design and amplification conditions validated by the manufacturer to ensure balanced detection across different pathogen types.

Although we cannot completely exclude the possibility of stability differences, we believe that through timely low-temperature storage and regular testing, we have

minimized its impact on our conclusions.

(2) Regarding bacterial colonization and copy number:

Your point about distinguishing true infection from colonization—particularly for common respiratory commensals—is critically important. As described in our response to the editor's related comments, we employed a comprehensive clinical adjudication framework to address this challenge. For bacteria detected in throat swabs, we applied stricter criteria requiring:

- High read counts or predominance within the microbial community;
- Strong clinical and radiographic evidence of infection;
- Reasonable exclusion of alternative pathogens.

We agree that focusing on copy number is meaningful, and our read count threshold (≥10 for bacteria) serves as a proxy indicator. In Supplementary Table 3, we further demonstrate that co-detections involving specific bacteria (Streptococcus pneumoniae, Haemophilus influenzae) were associated with significantly worse clinical outcomes, supporting their clinical relevance as true pathogens rather than mere colonization.

Regarding host RNA quality control (e.g., GAPDH):

We sincerely appreciate this excellent suggestion. Currently, our tNGS workflow incorporates exogenous internal reference nucleic acids to monitor the entire experimental process, including nucleic acid extraction, reverse transcription, amplification, and sequencing. This enables us to assess batch-to-batch consistency and identify failed experimental runs.

However, we agree that detecting host housekeeping genes such as GAPDH or β-actin would provide another valuable metric for sample quality—particularly regarding RNA integrity and the presence of respiratory epithelial cells. We will seriously consider incorporating this approach in our future studies to further strengthen sample quality assurance.

Thank you again for your insightful suggestions, which have helped us identify opportunities to refine our methodology.

Reviewer 3:

1. See attached file, as follows:

Journal: Microbiology Spectrum

Type of manuscript: Research Article

Manuscript ID: Spectrum00275-26

Title: High Prevalence and Clinical Impact of Microbial Co-detection in Hospitalized Children with Human Parainfluenza Virus Type 1

Summary: This manuscript describes a large retrospective study of clinical and laboratory records obtained from pediatric patients hospitalized with ARI at a single hospital in Nanning, Guangxi, China, from April 2021 to Dec 2024. Case enrollment required available test results from a large panel of respiratory pathogens using next generation sequencing of preamplified targets (tNGS). Analysis of 10,153 pediatric patients yielded 9,394 children that met specified selection criteria. Of these, HPIV-1 was detected in 164 (1.75%) by tNGS. Demographic, epidemiological and clinical data of HPIV-1 patients were analyzed and those with discrete and multiple pathogen infections compared. The authors concluded that these findings will advance efforts to develop algorithms that predict clinical progression of HPIV-1 ARI. The study findings are confirmatory rather than novel and it remains a question how these data can be used to build accurate predictive models.

Major concerns:

1) Detection methodology. The tNGS detection methodology used in this study appears flawed. No SARS-CoV-2 was included in the panel(s), despite it being an important cause of ARI prevalent during the study period. Also, it is inconceivable that HPIV-1 would be the most prevalent pathogen detected in this demographic, suggesting tNGS targeting bias or HPIV-1 target contamination. Moreover, the authors report that an “updated” manufacturers respiratory pathogen panel was introduced “during the study period”. All samples must be tested by one or the other panel, which is to say that the data from different panel tests cannot be combined

unless confirmed that identical test results would be obtained.

Response: Thank you for your insightful and constructive comments regarding the detection methodology. We appreciate the opportunity to clarify these important points.

(1) Regarding the absence of SARS-CoV-2 in the tNGS panel

We acknowledge that the initial tNGS panel (Table S1), used at the beginning of the study, did not include SARS-CoV-2. However, as stated in the manuscript, an updated manufacturer's respiratory pathogen panel (Table S2) was introduced during the study period, which does include SARS-CoV-2 ("Severe Acute Respiratory Syndrome Coronavirus 2").

Importantly, it is essential to contextualize the study period (April 2021 to December 2024) within China's unique public health measures. From 2020 to early 2023, strict COVID-19 containment policies were implemented nationwide. During this period, all hospitalized children with acute respiratory infection (ARI) were required to undergo routine SARS-CoV-2 RT-PCR testing as a mandatory admission screening measure. Only those who tested negative for SARS-CoV-2 and met the clinical indications for tNGS were subsequently enrolled for tNGS testing. This sequential testing algorithm explains why SARS-CoV-2 was not detected by tNGS in our cohort—patients with SARS-CoV-2 infection were systematically excluded prior to tNGS testing, rather than being missed due to panel limitations.

(2) Regarding the prevalence of HPIV-1

We apologize for any ambiguity in our original presentation. To clarify: HPIV-1 was not the most prevalent pathogen detected overall in our ARI cohort. Among the 9,394 cases that met the inclusion criteria, the pathogens with the highest detection rates were Haemophilus influenzae and Mycoplasma pneumoniae (each approximately 30%). In contrast, HPIV-1 was detected in only 1.7% (164/9,394) of eligible patients. The focus of this manuscript is specifically on children with HPIV-1 infection, examining their co-detection patterns and clinical characteristics.

(3) Regarding the use of different tNGS panels during the study period

We fully agree with the reviewer that data obtained from different diagnostic panels

cannot be combined without demonstrating comparability. We appreciate this important methodological concern.

Importantly, the core assay workflow, including target amplification primers and protocols, remained identical across all panel versions. The only difference was that the updated panels expanded to include additional, less common pathogens compared to the initial panel (65 targets).

To rigorously address the concern of data comparability, we performed a sensitivity analysis restricted to a "core microbe panel." This core panel consists of 64 targeted respiratory agents that were consistently included in all versions of the tNGS assay throughout the entire study period.

This analysis yielded similar overall co-detection rates compared to the original full-panel analysis: 94.5% versus 95.7%, with an absolute difference of only 1.2%. Since HPIV-1 was present in all panel versions, its detection rate remained unchanged in this sensitivity analysis.

These findings indicate that the expansion of the panel to include additional, less common microbes did not materially influence the study's primary findings regarding HPIV-1 detection and co-detection patterns. We have now incorporated this sensitivity analysis into the revised Results sections **(Manuscript, Lines 200–205, Page 9)**.

We believe these revisions comprehensively address the reviewer's concerns and significantly strengthen the methodological rigor of our study. We are grateful for the careful review, which has substantially improved our manuscript.

2. Study design. The retrospective study design using a single time-point specimen collection/testing of non-standard specimen types (where specimen type may correlate with patient age and therefore influence specific pathogen types detected) without agematched controls compromises clinical outcome prediction. The few patients with mono-HPIV-1-detections (7) further complicates comparisons with co-detection data (Lines 209-218, Table 2). This is a particular problem where age distribution bias between the two groups could explain differences in the measured variables (children under one year of age would be expected to have fewer

codetections because respiratory pathogen burden is directly correlated with age).

Response: We sincerely thank the reviewer for the insightful comments regarding the study design and potential biases. We fully acknowledge the limitations of this study, including its retrospective single-center design, the lack of age-matched controls, and the small number of HPIV-1 mono-detected cases (n = 7). To address these concerns, we conducted additional stratified analyses by age and specimen type (Supplementary Tables 4 and 5) and have updated the Results section accordingly **(Manuscript, Lines 215–230, Page 10)**.

①Correlation between specimen type and patient age

As shown in Supplementary Table 4, specimen type selection was indeed associated with age: BALF was more frequently collected in children aged >12 months than in those ≤12 months (62.9% vs. 44.1%, P=0.020). However, the detection rates of the three most common pathogens co-detected with HPIV-1—Haemophilus influenzae, Streptococcus pneumoniae, and human rhinovirus—were comparable across BALF, throat swab, and sputum specimens (all P>0.05). This consistency across specimen types suggests that age-related differences in specimen collection had limited impact on our co-detection analysis and minimized concerns regarding detection bias.

②Age distribution bias

We agree with the reviewer that age is a critical confounder—younger infants naturally have a lower respiratory pathogen burden and therefore exhibit lower rates of co-detection. To rigorously address this issue, we performed an age-stratified subgroup analysis (≤12 months vs. >12 months), as presented in Table S5. The key findings are as follows:

- In the ≤12 months subgroup (mono-detection: n=5; co-detection: n=54), children with co-detection had significantly longer hospital stays (7.0 vs. 5.0 days, P=0.006) and higher treatment expenses (9194.1 vs. 3358.0 CNY, P=0.009) compared to those with mono-detection.
- In the >12 months subgroup (mono-detection: n=2; co-detection: n=98), no statistically significant differences were observed in hospital stay or treatment costs (both P>0.05). However, the extremely small sample size of the

mono-detection group (n=2) in this subgroup severely limits the reliability of any comparative conclusions regarding co-detection outcomes in older children.

These age-stratified results demonstrate that, even in the ≤12 months age group—where baseline co-detection rates are naturally lower—HPIV-1 co-detection remained consistently associated with more severe clinical outcomes. This supports the conclusion that the association between co-detection and disease severity is independent of age and does not merely reflect age-related differences in pathogen exposure.

③Limitations and future directions

We have incorporated these limitations into the revised Discussion section and proposed that future prospective multicenter studies with standardized specimen collection protocols and larger sample sizes are needed to validate our findings **(Manuscript, Lines 369–374, Page 16)**.

In summary, while we acknowledge the inherent limitations of this retrospective study, the significant findings observed in the ≤12 months subgroup—combined with the consistency of pathogen detection rates across specimen types—support the conclusion that HPIV-1 co-detection is independently associated with worse clinical outcomes in children with acute respiratory infection. These additional analyses have substantially strengthened the rigor of our study, and we are grateful to the reviewer for prompting these improvements.

3. Infection vs. colonization. The contribution of "potential pathogens to the ARI and overall clinical presentation" (Lines 148-163) cannot be accurately established for most of these pathogens by the proposed methodology. Establishing a pathogen's role in disease requires additional information; e.g., pathogen load, measured changes in pathogen load, serology to establish acute infection or pathology of affected organs/tissues. The attributable contribution to disease with multiple pathogen detections would be even more difficult to establish.

Response: We thank the reviewer for raising this important point regarding the challenge of distinguishing infection from colonization in tNGS-based studies. We fully

acknowledge that detecting a microorganism does not equate to establishing its causative role in disease—particularly in cases of multi-pathogen co-detection—and that definitive attribution typically requires complementary evidence such as pathogen load dynamics, serology, or histopathology.

To address this concern and minimize its impact on our findings, we implemented the following measures in the study design and interpretation:

①Strict inclusion criteria. We enrolled only symptomatic hospitalized children with a confirmed clinical diagnosis of acute respiratory infection (ARI). Asymptomatic individuals, in whom colonization is more likely, were excluded from the study population.

②Integration of clinical and laboratory data. tNGS results were not interpreted in isolation. Instead, they were systematically evaluated alongside clinical manifestations, inflammatory biomarkers (e.g., C-reactive protein, procalcitonin), and radiographic findings to support the distinction between true infection and incidental colonization.

③Pathogen-specific caution in interpretation. For pathogens with high colonization potential in the respiratory tract (e.g., Streptococcus pneumoniae, Haemophilus influenzae), we exercised particular caution in attributing causality, especially in the absence of consistent clinical or laboratory evidence of active infection.

We have incorporated this discussion into the Limitations section of the revised manuscript **(Manuscript, Lines 364–367, Page 16)**. We also acknowledge that future prospective studies incorporating quantitative culture, serological assays, or longitudinal pathogen load monitoring are needed to further validate and refine our findings.

We thank the reviewer for highlighting this critical issue, which has helped us strengthen the rigor and transparency of our manuscript.

4. Why were older children excluded from the study (enrolled aged ≤14 years)? The internationally accepted definition of a child is generally any person under the age of 18.

Response: We thank the reviewer for this thoughtful comment regarding the age range of our study population. We fully agree that the internationally accepted definition of children generally includes individuals under 18 years of age.

In the present study, we restricted enrollment to children aged ≤14 years based on the following considerations:

① Epidemiological and clinical relevance. Acute respiratory infections (ARIs) disproportionately affect young children, with the highest incidence and hospitalization rates occurring in those under 5 years of age. The burden of ARI decreases substantially after early adolescence. In our study cohort, over 95% of HPIV-1-positive cases were under 14 years of age, and only a negligible number of patients aged 15–17 years were admitted with ARI during the study period. Thus, the ≤14 years cutoff captures the vast majority of the pediatric ARI population while maintaining clinical homogeneity.

②Consistency with prior pediatric respiratory infection research. Numerous previous studies on respiratory infections in children have adopted a ≤14 years age cutoff to define the pediatric population [1-4]. This convention facilitates cross-study comparisons and aligns with routine pediatric practice in many clinical settings, including our institution.

③Generalizability to older adolescents. We acknowledge that excluding individuals aged 15–17 years may limit the generalizability of our findings to this older subgroup. However, given the very low incidence of HPIV-1-associated ARI hospitalization in this age group, the impact on our conclusions is likely minimal. Future multicenter studies with broader age coverage would help validate our findings across the full pediatric age spectrum.

We thank the reviewer again for raising this important point, which has helped us clarify the rationale for our study design. The references are as follows:

[1] Human parainfluenza virus types 1-4 in hospitalized children with acute lower respiratory infections in China.

[2] Intraday effects of outdoor air pollution on acute upper and lower respiratory infections in Australian children.

[3] A novel combined nomogram for predicting severe acute lower respiratory tract infection in children hospitalized for RSV infection during the post-COVID-19 period.

[4] Newly identified respiratory viruses associated with acute lower respiratory tract infections in children in Lanzou, China, from 2006 to 2009.

5. Do not present data out to 3 decimal places, as this is unsupportable accuracy (example, Table 2).

Response: Thank you for your comment. We have revised all numerical data in the manuscript and Table 2 to one decimal place as suggested.

6. Be consistent with italicizing genus/species names (e.g., Supplementary Tables 1 &2; Figure 2). Try to consolidate names where appropriate (e.g., parainfluenza virus types 1,2,3).

Response: Thank you for the valuable suggestion. We have corrected the italicization of all genus and species names throughout the manuscript, including Supplementary Tables 1 and 2 and Figure 2, to ensure consistency.We have also consolidated related pathogen names where appropriate for better readability.

7. Figure 1. Exclusion should be “>14 years”.

Response: Thank you for this correction. We have revised the exclusion criterion in Figure 1 to “>14 years”.

8. Line 186. “Males were significantly more prevalent than females”. This finding for HPIV-1 patients cannot be interpreted without knowing what the proportion of males and females was in the overall study sample.

Response: We thank the reviewer for this important observation. We agree that the gender distribution of HPIV-1-positive patients should be interpreted in the context of the overall study population.

In the original manuscript, we reported that males were significantly more prevalent among HPIV-1-positive children (61.0% vs. 39.0%; P<0.05). Following the reviewer’s

comment, we re-evaluated this finding alongside the gender composition of the total study sample. The overall study population (N=9,394) consisted of 60.1% males and 39.9% females, which is comparable to the gender distribution observed in the HPIV-1-positive subgroup.

To address this concern, we have revised the relevant sentence in the manuscript **(Manuscript, Lines 180–182, Page 8)** as follows:

"The gender distribution among these children (60.98% male, 39.02% female) was consistent with that of the overall study population (60.1% male, 39.9% female)."

This revision clarifies that the male predominance observed in HPIV-1-positive cases reflects the baseline gender composition of the study cohort rather than a specific predisposition to HPIV-1 infection.

We thank the reviewer again for helping us improve the accuracy and interpretability of our results.

9. Lines 198, 201, 296. The term “microorganisms” suggests all respiratory tract flora when only those associated with ARI were tested (respiratory pathogens).

Response: We thank the reviewer for this careful and important comment. We agree that the term “microorganisms” is overly broad, as it may misleadingly imply testing of all respiratory tract flora, whereas only a predefined panel of ARI-associated agents was targeted. We also acknowledge that detection does not equate to causation. To address this, we have replaced “microorganisms” with “targeted respiratory agents” in lines 198, 201, and 296 of the original manuscript **(Manuscript, Lines 189, 192, Page 8; Line 306, Page 13)**.

10. Lines 281-282. Peak circulation of HPIV-1 occurs during odd-numbered years in temperate regions which was not demonstrated in this study.

Response: We thank the reviewer for this insightful comment. We agree that HPIV-1 typically exhibits a biennial circulation pattern with peak activity in odd-numbered years in temperate regions. However, our study period (2021–2024) overlapped with the COVID-19 pandemic, during which non-pharmaceutical interventions (e.g.,

masking, social distancing) and shifts in population immunity profoundly disrupted the expected seasonality and biennial cycles of common respiratory viruses. This disruption likely explains the absence of the typical odd-year peak of HPIV-1 in our cohort. We have incorporated this explanation into the second paragraph of the Discussion section **(Manuscript, Lines 292–295, Page 13)**.

Reviewer 4:

This study describes the analysis of human parainfluenza virus type 1 infection in a large cohort (~9,000) of hospitalized children, utilizing next-generation sequencing to reveal the prevalence of HPIV-1 and the presence of viral and bacterial co-infections. The manuscript is well-written and despite the reliance on NGS, which will detect commensal pathogens, the authors combine their findings with in depth clinical analysis that is well laid out and comprehensive to ensure only patients with active infections are identified. The findings are clear and well-presented and offer a new tool for clinicians to understand the prevalence and symptoms of HPIV-1 infection in different age groups. Important findings include the age group difference in prevalence of infection and co-infection and the difference in hospitalization rates. Overall, the manuscript provides a robust and methodologically modern look at pediatric acute respiratory infection due to HPIV-1.

This manuscript is very interesting and useful for the field. My only minor critique is to remove statements on antibody titers increasing with infection as it does not appear these were tested in a way where antigen-specificity is known against HPIV-1.

Response: We sincerely thank the reviewer for the positive feedback and the thoughtful comment. We agree that it is important to avoid any potential misinterpretation that the observed differences in immunoglobulin levels reflect HPIV-1-specific antibody responses, as only total serum IgG, IgA, and IgM were measured in this study. Accordingly, we have revised the relevant descriptions in both the Results **(Manuscript, Lines 210–211, Page 9)** and Discussion sections **(Manuscript, Lines 317–319, Page 14)** to explicitly clarify that these are total

immunoglobulin levels, not HPIV-1-specific antibody titers. We believe this clarification effectively addresses the concern.

Reviewer 5:

In this study, the authors characterized the epidemiology, microbial co-detection profiles, and associated clinical features of HPIV-1 in hospitalized children with acute respiratory infections using targeted next-generation sequencing. This is a well-conducted and clearly written study, and the conclusions are supported by the data. I have only a few minor comments for the authors' consideration.

1. The abstract is clear but includes many numbers, especially in the Results section. The authors may consider reducing the number of details and focusing on the main findings to improve readability.

Response: We thank the reviewer for the positive feedback and the constructive suggestion. In accordance with the recommendation, we have revised the Abstract to improve readability by reducing the number of specific numerical details and focusing more on the main findings. The updated Abstract now presents key results in a more concise and summarized manner, while retaining the essential conclusions. We believe this revision enhances clarity and aligns with the reviewer's valuable suggestion.

2. A brief statement describing HPIV as an RNA virus in the family Paramyxoviridae may help non-specialist readers.

Response: We thank the reviewer for this helpful suggestion. To assist non-specialist readers, we have added a brief introductory statement describing the virological characteristics of human parainfluenza virus (HPIV) at the beginning of the Introduction section **(Manuscript, Lines 62–67, Page 3)**. The revised text now reads as follows:

"According to the first global burden estimates for acute lower respiratory infections in children, human parainfluenza virus (HPIV) — an enveloped, negative-sense

single-stranded RNA virus belonging to the family Paramyxoviridae—is responsible for approximately 13% of all pediatric acute lower respiratory infection cases and is associated with 4% to 14% of related hospitalizations [1], thereby contributing substantially to the healthcare burden."

We believe this addition improves the accessibility of the manuscript for a broader audience while maintaining scientific accuracy.

We hope that these changes address the concerns raised and that the revised manuscript meets your approval. Thank you again for your thoughtful and thorough review of our work.

Sincerely,

Jie Tan

Pediatric Respiratory Medicine

The Maternal and Child Health Hospital of Guangxi Zhuang Autonomous Region

No.59 Xiangzhu Road

Nanning 530003

Guangxi Zhuang Autonomous Region, P. R. China

Fax: (+86-771)-2860 792

E-mail: tanjie273@163.com

Re: Spectrum00275-26R1 (**High Prevalence and Clinical Impact of Microbial Co-detection in Hospitalized Children with Human Parainfluenza Virus Type 1**)

Dear Dr. Jie Tan:

Thank you for the privilege of reviewing your work. Below you will find my comments, instructions from the Spectrum editorial office, and the reviewer comments.

Please return the manuscript within 60 days; if you cannot complete the modification within this time period, please contact me. If you do not wish to modify the manuscript and prefer to submit it to another journal, notify me immediately so that the manuscript may be formally withdrawn from consideration by Spectrum.

Revision Guidelines
To submit your modified manuscript, log into the submission site at https://spectrum.msubmit.net/cgi-bin/main.plex. Go to Author Tasks and click the appropriate manuscript title to begin. The information you entered when you first submitted the paper will be displayed; update this as necessary. Note the following requirements:

• Upload point-by-point responses to the issues raised by the reviewers in a file named "Response to Reviewers," NOT in your cover letter.
• Upload a compare copy of the manuscript (without figures) as a "Marked-Up Manuscript" file.
• Upload a clean .DOC/.DOCX version of the revised manuscript and remove the previous version.
• Each figure must be uploaded as a separate, editable, high-resolution file (TIFF or EPS preferred), and any multipanel figures must be assembled into one file.
• Any supplemental material intended for posting by ASM should be uploaded with their legends separate from the main manuscript. You can combine all supplemental material into one file (preferred) or split it into a maximum of 10 files with all associated legends included.

For complete guidelines on revision requirements, see our Submission and Review Process webpage. Submission of a paper that does not conform to guidelines may delay acceptance of your manuscript.

Data availability: ASM policy requires that data be available to the public upon online posting of the article, so please verify all links to sequence records, if present, and make sure that each number retrieves the full record of the data. If a new accession number is not linked or a link is broken, provide Spectrum production staff with the correct URL for the record. If the accession numbers for new data are not publicly accessible before the expected online posting of the article, publication may be delayed; please contact production staff (Spectrum@asmusa.org) immediately with the expected release date.

Publication Fees: For information on publication fees and which article types are subject to charges, visit our website. If your manuscript is accepted for publication and any fees apply, you will be contacted separately about payment during the production process; please follow the instructions in that e-mail. Arrangements for payment must be made before your article is published.

ASM Membership: Corresponding authors may join or renew ASM membership to obtain discounts on publication fees. Need to upgrade your membership level? Please contact Customer Service at Service@asmusa.org.

The ASM Journals program strives for constant improvement in our submission and publication process. Please tell us how we can improve your experience by taking this quick Author Survey.

Thank you for submitting your paper to Spectrum.

Sincerely,
Yuan Pin Hung
Editor
Microbiology Spectrum

Reviewer #3 (Comments for the Author):

Spectrum00275-26R1
Reviewer #3 Comments

1) The "sequential testing algorithm" (Figure 1) does not indicate the exclusion of SARS-CoV-2 positive patients. The exclusion

of SARS-CoV-2 from this ARI cohort should be explicitly stated in the Materials and Methods, Inclusion and Exclusion Criteria section.

2) HPIV-1 was the most common viral pathogen detected in the ARI case cohort, 4x the next most common virus, rhinovirus. This is unlikely in this age and clinical cohort, where rhinovirus or other respiratory viruses would more likely be detected. The exclusion of SARS-CoV-2 from the cohort cannot explain this. The distortion of normal circulation patterns of respiratory viruses by SARS-CoV-2 as documented elsewhere might account for this, but this needs to be part of the Discussion.

3) The Authors responses and manuscript changes to other Reviewer #3 comments are acceptable.

Reviewer #5 (Comments for the Author):

The authors have appropriately revised the manuscript in response to the reviewers' comments.

Apr 3, 2026

Editorial Board

Microbiology Spectrum

Dear Editor,

We greatly appreciate the valuable comments provided by the reviewer, which have significantly contributed to improving our manuscript **“High Prevalence and Clinical Impact of Microbial Co-detection in Hospitalized Children with Human Parainfluenza Virus Type 1”** (Spectrum00275-26R1). We have revised the manuscript accordingly and **highlighted in red in the updated version**. Our responses to the reviewer's comments are outlined below.

Reviewer #3 Comments:

1. The "sequential testing algorithm" (Figure 1) does not indicate the exclusion of SARS-CoV-2 positive patients. The exclusion of SARS-CoV-2 from this ARI cohort should be explicitly stated in the Materials and Methods, Inclusion and Exclusion Criteria section.

Response: Thank you for your thoughtful comment.

We would like to clarify that SARS-CoV-2 positive patients were not excluded from this ARI cohort. Our rationale is as follows:

1) Study period and evolving pandemic context: The study period (April 2021 to December 2024) spanned both the COVID-19 pandemic and post-pandemic phases, during which national testing policies varied. During the pandemic phase (2020–early 2023), China implemented strict COVID-19 control measures. All children hospitalized with ARI were required to undergo routine SARS-CoV-2 RT-PCR testing upon admission. Only those who tested negative by RT-PCR and met clinical indications for tNGS were subsequently enrolled for tNGS. After the pandemic phase, children hospitalized with ARI who met tNGS criteria were directly enrolled, regardless of SARS-CoV-2 status.

2) SARS-CoV-2 detection by tNGS: Among the 9,394 cases that met the inclusion criteria, tNGS detected SARS-CoV-2 in approximately 145 cases, all identified between May 2023 and December 2024.

To address the reviewer's concern and improve clarity, we have added the following statement to the Materials and Methods section **(Manuscript, Lines 135–138, Page 6)**:

"During the COVID-19 pandemic phase, all children hospitalized with ARI were required to undergo routine SARS-CoV-2 RT-PCR testing upon admission. Only those with negative RT-PCR results who also met clinical indications for tNGS were enrolled for tNGS testing."

We believe this revision adequately addresses the reviewer's comment.

2. HPIV-1 was the most common viral pathogen detected in the ARI case cohort, 4x the next most common virus, rhinovirus. This is unlikely in this age and clinical cohort, where rhinovirus or other respiratory viruses would more likely be detected. The exclusion of SARS-CoV-2 from the cohort cannot explain this. The distortion of normal circulation patterns of respiratory viruses by SARS-CoV-2 as documented elsewhere might account for this, but this needs to be part of the Discussion.

Response: Thank you for your valuable comment. We apologize for any lack of clarity in our original manuscript and would like to provide further clarification.

First, in our ARI cohort, HPIV-1 was not the most commonly detected pathogen in the overall study population. Among the 9,394 patients who met the inclusion criteria, the top five detected microorganisms were *Haemophilus influenzae*, *Mycoplasma pneumoniae*, rhinovirus, *Streptococcus pneumoniae*, and cytomegalovirus (each with detection rates >20%). In contrast, the detection rate of HPIV-1 in this eligible cohort was only 1.7% (164/9,394).

Second, the focus of our study was specifically on HPIV-1-positive children, investigating their co-detection patterns and clinical characteristics. The co-detection patterns presented in the manuscript reflect the co-occurrence of other pathogens

among HPIV-1-positive cases. Among the 164 HPIV-1-positive children, the top five co-detected microorganisms were *Haemophilus influenzae*, *Streptococcus pneumoniae*, rhinovirus, *Mycoplasma pneumoniae*, and cytomegalovirus (each with co-detection rates >23%). Notably, these five microorganisms also ranked among the top five most frequently detected pathogens in the overall eligible cohort of 9,394 patients.

We hope this clarification adequately addresses the reviewer's concern.

We hope that these changes address the concerns raised and that the revised manuscript meets your approval. Thank you again for your thoughtful and thorough review of our work.

Sincerely,

Jie Tan

Pediatric Respiratory Medicine

The Maternal and Child Health Hospital of Guangxi Zhuang Autonomous Region

No.59 Xiangzhu Road

Nanning 530003

Guangxi Zhuang Autonomous Region, P. R. China

Fax: (+86-771)-2860 792

E-mail: tanjie273@163.com

Re: Spectrum00275-26R2 (**High Prevalence and Clinical Impact of Microbial Co-detection in Hospitalized Children with Human Parainfluenza Virus Type 1**)

Dear Dr. Jie Tan:

Your manuscript has been accepted, and I am forwarding it to the ASM production staff for publication. Your paper will first be checked to make sure all elements meet the technical requirements. ASM staff will contact you if anything needs to be revised before copyediting and production can begin. Otherwise, you will be notified when your proofs are ready to be viewed.

Data Availability: ASM policy requires that data be available to the public upon online posting of the article, so please verify all links to sequence records, if present, and make sure that each number retrieves the full record of the data. If a new accession number is not linked or a link is broken, provide production staff with the correct URL for the record. If the accession numbers for new data are not publicly accessible before the expected online posting of the article, publication may be delayed; please contact ASM production staff immediately with the expected release date.

Publication Fees: For information on publication fees and which article types have charges, please visit our website. We have partnered with Copyright Clearance Center (CCC) to collect author charges. If fees apply to your paper, you will receive a message from no-reply@copyright.com with further instructions. For questions related to paying charges through RightsLink, please contact CCC at ASM_Support@copyright.com or toll free at +1-877-622-5543. CCC makes every attempt to respond to all emails within 24 hours.

ASM Membership: Corresponding authors may join or renew ASM membership to obtain discounts on publication fees. Need to upgrade your membership level? Please contact Customer Service at Service@asmusa.org.

Embargo Policy: A press release may be issued as soon as the manuscript is posted on the Spectrum Latest Articles webpage. The corresponding author will receive an email with the subject line "ASM Journals Author Services Notification" when the article is available online.

The ASM Journals program strives for constant improvement in our submission and publication process. Please tell us how we can improve your experience by taking this quick Author Survey.

Thank you for submitting your paper to Spectrum.

Sincerely,
Yuan Pin Hung
Editor
Microbiology Spectrum

Reviewer #3 (Comments for the Author):

Reviewer #3 response to Authors revisions.

1) Authors revision is acceptable.
2) Figure 2, Panel D. The legend states, "Top 10 detected viruses ...". The first histogram bar shows the total HPIV-1 detections (164) followed by bars showing detections of other respiratory pathogens that are co-detections. Panel D mixes data types and does not clarify this in the Legend or the Y-axis label. This is confusing and needs to be corrected. Also, the Panel E legend states "Top 21 co-detected targeted respiratory agents ', yet 22 agents are listed.

Reviewer #5 (Comments for the Author):

The authors have appropriately revised the manuscript in response to the reviewers' comments.
